# Conservation of Intronic Sequences in Vertebrate Mitochondrial Solute Carrier Genes (Zebrafish, Chicken, Mouse and Human)

**DOI:** 10.3390/ncrna5010004

**Published:** 2019-01-06

**Authors:** Rosa Calvello, Antonia Cianciulli, Vincenzo Mitolo, Annalisa Porro, Maria Antonietta Panaro

**Affiliations:** 1 Department of Biosciences, Biotechnologies and Biopharmaceutics, University of Bari, via Orabona, 4, I-70126 Bari, Italy; rosa.calvello@uniba.it (R.C.); antonia.cianciulli@uniba.it (A.C.); vmitolo@alice.it (V.M.); 2 Department of Clinical and Experimental Medicine, University of Foggia, I-71122 Foggia, Italy; chiara.porro@unifg.it

**Keywords:** mitochondrial carriers, introns, human, mouse, chicken, zebrafish

## Abstract

The conservation of intronic sequences was studied in the mitochondrial solute carrier (SLC25A*) genes of Zebrafish, Chicken, Mouse and Human. These genes are homologous and the coding sequences have been well conserved throughout Vertebrates, but the corresponding intronic sequences have been extensively re-edited. However, significant segments of Zebrafish introns are conserved in Chicken, Mouse and Human in carriers SLC25A3, SLC25A21, SLC25A25, SLC25A26, and SLC25A36; Chicken intron segments are conserved in Mouse or Human in three additional carriers, namely SLC25A12, SLC25A13, and SLC25A29. Thus, a quota of the intronic sequences of Euteleostomi has been transferred (through Sarcopterygii) to Birds and (through Sarcopterygii and ancestral Mammals) to Mouse and Human. The degree of conservation of Euteleostomi-derived sequences is low and quite similar in Chicken, Mouse and Human (0.23–0.27%). The overall degree of conservation of Sarcopterygii-derived sequences in Mammals is higher, and it is significantly higher in Human than in Mouse (4.4% and 3.2%, respectively). Some of the conserved intronic sequences of SLC25A3, SLC25A21, SLC25A25, and SLC25A29 are exonized in some transcript variants of Zebrafish, Chicken, Mouse, and Human and, with minor nucleotide changes, in other Birds or Mammals.

## 1. Introduction

Nuclear pre-mRNA sequences of most protein-coding genes are characterized by the regular alternation of multiple exonic and intronic segments. The exonic segments are those eventually coding for a protein product, while the introns are non-coding segments. During the maturation of the final mRNA product, the introns are removed by spliceosomes in the process of RNA splicing. The non-coding introns, once considered junk DNA, are now believed to be involved in gene expression regulation by biological processes, which, however, are still poorly understood.

Here we study the conservation of intronic sequences in the mitochondrial solute carrier (SLC25A*) genes of Zebrafish, Chicken, Mouse and Human. These genes are homologous and the coding sequences have been well conserved throughout Vertebrates, for mitochondria and the corresponding solute carriers assist in very basic biological functions which thereby have remained unchanged in vertebrate evolution [1,2]; indeed, little alterations of carrier structure may entail severe dysfunctions [3]. On the contrary, the non-coding introns have usually diverged considerably in their nucleotide composition. However, some intronic sequences were found to be conserved in Zebrafish, Chicken, Mouse, and Human in SLC25A3, SLC25A21, SLC25A25, SLC25A26, and SLC25A36 and other, more numerous, segments are conserved in Chicken, Mouse and Human only in SLC25A12, SLC25A13, and SLC25A29. In some of the transcripts of SLC25A3, SLC25A21, SLC25A25, and SLC25A29 conserved intronic sequences (CISs) were found to have been exonized.

## 2. Results

All the mitochondrial solute carriers genes of the species studied have been searched for Zebrafish/Chicken/Mouse/Human or Chicken/Mouse/Human CISs, but only the eight carriers mentioned above proved to harbor significant CISs and will be individually considered. The Supplementary Material Table S1 (Table S1) lists all the transcript variants studied.

### 2.1. SLC25A3

Two nucleotide sequences in the interval between Exon 1 and Exon 3 are conserved in Zebrafish (SLC25A3b), Chicken, Mouse and Human. Table S2 shows these sequences and their alignments. The upstream sequences are 119-nt long and the downstream sequences 126-nt long; in both sequences the conservation among the species is ≥71%. Furthermore, in each species the per cent nucleotide identity between the upstream and downstream sequences ranges between 84% (Zebrafish) and 66% (Mouse).

The two conserved nucleotide sequences possibly originated from the duplication, in Fish, of an originally single sequence and the duplication was conserved in Birds and Mammals. Both sequences might in principle be translated into a complete putative conserved *Mito_carr* domain, corresponding to the first transmembrane segment and the immediately adjoining cytoplasmic and mitochondrial matrix segments. Actually, only one of the two sequences is translated in Zebrafish, Chicken, Mouse and Human SLC25A3 proteins each time, while the other is spliced out; accordingly, these species exhibit two similar SLC25A3 variant transcripts. For a more detailed description and comments concerning other fish, amphibian, bird and mammalian species, see Calvello et al. [4].

A CIS present in Chicken, Mouse and Human only is displayed in Table S3. This segment remains untranslated in all three species. Note the more downstream-extended Chicken/Human conservation, as compared to the Chicken/Mouse conservation.

### 2.2. SLC25A12

No significant Zebrafish intronic sequence is conserved in Birds and Mammals, but three CISs are present in Chicken, Mouse and Human (see, as an example, Table S4). Note that, in this sequence, the Chicken/Human conservation is more extended both upstream and downstream than the Chicken/Mouse conservation. None of the above CIS sequences is translated in the transcript variants listed in Table S1. In addition, a further short untranslated CIS is present in Chicken and Mouse only.

### 2.3. SLC25A13

While no significant Zebrafish intronic sequence is conserved in Birds and Mammals, 12 CISs are present in Chicken, Mouse and Human (see, as an example, Table S5). Note that, in this specific sequence, the Chicken/Human conservation is more extended both upstream and downstream than the Chicken/Mouse conservation. This is the more usual arrangement, although in other instances the Chicken/Mouse/Human alignments have equal lengths and in a few cases the Chicken/Mouse alignments are somewhat more extended than the Chicken/Human. None of the above sequences is translated in the transcript variants listed in Table S1.

In addition, four untranslated CISs are present in Chicken and Human only.

### 2.4. SLC25A21

This “gigantic” gene (more than 200,000 nt in Zebrafish and almost half a million nt in Human) is the one that exhibits the highest number of conserved intronic sequences. Four sequences are conserved in Zebrafish, Chicken, Mouse and Human; one sequence is conserved in Zebrafish, Chicken and Human; one sequence is conserved in Zebrafish and Human only. In addition, 33 sequences are conserved in Chicken, Mouse and Human and 10 sequences in Chicken and Human only.

All four Zebrafish/Chicken/Mouse/Human alignments are shown in Tables S6–S9. The longest alignment is displayed in Table S6: here 364 nt of Zebrafish align with 332 nt of Chicken, 274 nt of Mouse and 243 nt of Human; the section exhibiting the alignment of all four species is 241 nt long.

The longest (656 nt) Chicken/Mouse/Human alignment is shown in Table S10. Note that the alignment is very “compact” with relatively few gaps.

In several instances the Chicken/Human alignment exceeds the Chicken/Mouse alignment upstream or downstream or at both sites, as in the example shown in Table S11.

In total, the nucleotides aligning with the intronic Zebrafish SLC25A21 sequence number 786 in Chicken, 690 in Mouse and 784 in Human. There are 10,111 nucleotides aligning with the intronic Chicken SLC25A21 sequence in Mouse and 12,918 in Human.

In total, 786 Chicken nucleotides, 690 Mouse nucleotides and 784 Human nucleotides align with the intronic Zebrafish SLC25A21 sequence; 10,111 Mouse nucleotides and 12,918 Human nucleotides align with the intronic Chicken SLC25A21 sequence.

Notably, none of these CISs is translated in any of the Zebrafish, Chicken, Mouse or Human SLC25A21 proteins listed in Table S1. However, some of these CISs with few nucleotide alterations can be exonized in other Bird or Mammalian species.

A sequence very similar to a section of the SLC25A21 Human intronic sequence is transcribed in the SLC25A21 of Gorilla (XM_004055094.1 and XP_004055142.1) first exon (Table S12). In the Table, the aligned Human/Gorilla sequences are underlined and in bold type; these are identical except for a single nucleotide. The translations (frame +1)

Human MAYPAACLGGSAFHDRHSLLRIVGSGSGLQLQITAAVLR*
Gorilla MAYPAACLGGSAFHDRHSLLRIVGSGSGLQLQITAAVLRL

reveal, in Human, a stop codon TAA (boxed) which is skipped in Gorilla by a mutation of the triplet into TTA (boxed). In addition, Chicken and Mouse have stop codons at the same position (boxed).

Sequences very similar to a section of the SLC25A21 Chicken intronic sequence are transcribed in the SLC25A21 variant X1 of *Anas platyrhynchos* (mallard; XM_013107923.1; XP_012963377.1) and SLC25A21 variant X4 of *Zonotrichia albicollis* (white-throated sparrow; XM_014268755.1; XP_014124230.1) first exons (Table S13). First exons of the variants X2 (XM_013107924.1; XP_012963378.1) and X3 (XM_013107925.1; XP_012963379.1) of the SLC25A21 of *Anas platyrhynchos* are identical to that of variant X1.

The translations (frame +3)

Chicken PIKWLTDELWGRFILFMHSQTSCFKHIFLP*CY*DI
Anas PIQWLTDELLGQIYIVRAFPDILFQAYIFTLELLRHL
Zonotrichia PIQWLTDELLGQIYIAHVFPDILFQAYIFILALLRHL

reveal the occurrence of stop codons in Chicken, which are skipped in Anas and Zonotrichia. The differences in the reading frames are due to the presence, in Anas and Zonotrichia, of an additional nucleotide (boxed in the alignment) which is lacking in Chicken (as well as in Mouse and Human).

A sequence very similar to a section of the SLC25A21 Chicken, Mouse and Human intronic sequences is transcribed in the SLC25A21 of *Physeter catodon* (sperm whale; XM_007105733.1; XP_007105795.1) first exon (Table S14). In the Table the aligned Chicken, Mouse, Human and Physeter sequences are underlined and in bold type. The translations (frame +1)

Chicken MCKNIANRIVKKTSSSIGNPNVFR*NNM
Mouse MCKNIANGIVKKTSSSLGNPNVLGWNNR
Human MCKNIANGIVKKTSSSLGNPNVFRWNNR
Physeter MCKNIANGIVKKTSSSLGNPNVFRWNNR

show that in the Chicken the translation is interrupted by the stop codon TAA (boxed), which corresponds to TGG in Mouse, Human and Physeter. Thus, the Mouse and the Human sequences *could, in theory, be translated* as in Physeter, where the ATG (position 481–483 in the alignment) is the start codon.

### 2.5. SLC25A25

One DNA sequence is conserved in Zebrafish (slc25a25-A), Chicken, Mouse and Human (Table S15) and another DNA sequence in Chicken, Mouse and Human only (Table S16).

Contrary to other intronic conserved sequences, the above sequences of the SLC25A25 genes are exonized in several Zebrafish (slc25a25-A), Chicken, Mouse and Human transcript variants.

The general composition of these gene transcripts is:

Exon 1 (transcription optional)
Exon 2 (transcription optional)
First conserved segment (Table S15)
Exon 3 (constant)
Exon 4 (constant)
Second conserved segment (Table S16)
6 or 7 Exons (constant)

Table S17 displays, in the blue boxes, the transcript components which are actually expressed in each variant. At the upstream end the transcript composition is very variable, while at the downstream end (after the Second conserved segment) in all transcripts there are seven exons, reduced to six in some Chicken transcripts, by fusion of two exons due to the transcription of the short intervening intron.

The Chicken transcripts X4 and X5, the Mouse transcripts 2 and 1 and the Human transcripts 2 and X1 are the “longest” transcripts, as all these express the optional Exon 1.

The Chicken transcripts X3, X1 and X7, the Mouse transcripts 3, X3, X1 and X4, and the Human transcripts 3 and 5, which do not express Exon 1, start with Exon 2 instead.

The Zebrafish transcript of slc25a25-A, the Chicken transcripts X2 and X6, the Mouse transcripts 4 and X2, and the Human transcripts 1 and X2, which do not express neither Exon 1 nor Exon 2, start with the First conserved segment instead. It should be remarked that in the Chicken transcripts X2 and X6 the First conserved segment is immediately preceded by and fused with a 117-nt segment which is not transcribed in all other variants (this segment is indicated in Table S17 by a narrower red box).

In brief, when Exon 1 is transcribed, Exon 2 and the First conserved segment are not transcribed. When Exon 1 is skipped, Exon 2 may provide the start codon and the First conserved segment is not transcribed. However, when neither Exon 1 nor Exon 2 are expressed, the start codon is provided by the First conserved segment. Only two exceptionally short Human transcripts (X3 and X4) lack not only Exon 1, Exon 2 and the First conserved segment, but also the otherwise constant Exon 3, and start with Exon 4.

The second, shorter, conserved segment is transcribed in nine of the Chicken, Mouse and Human transcripts. However, only the underlined sections of Table S16 are actually transcribed and make up a supernumerary isolated exon, bounded by a conserved AG upstream and a conserved GT downstream.

All Zebrafish (slc25a25-A), Chicken, Mouse and Human transcript variants exhibit the three canonical *Mito_carr* domains; in addition all transcripts, except the two shortened Human transcripts X3 and X4, exhibit an Ca^2+^ binding site.

The First conserved segment is always completely transcribed with +1 frame. The Second conserved segment is always transcribed with +2 frame. Both sequences contain no putative conserved domain.

### 2.6. SLC25A26

One CIS is present in Zebrafish and Chicken; 6 CISs are present in Chicken, Mouse and Human and 5 CISs in Chicken and Human only. None of the above sequences is translated in the transcript variants listed in Table S1.

### 2.7. SLC25A29

Only one DNA sequence is conserved in Chicken and Human (Table S18). Both the Chicken and the Human sequences, if translated, would be interrupted by stop codons in any frame. The conserved sequence is expressed only in the Human transcript variant X10, where it is read with frame + 1; the aa transcription is DVMLKFCVALTAFTF*, which corresponds to the bold underlined segment in Table S18. The truncated protein exhibits no *Mito_carr* domain.

### 2.8. SLC25A36

There is one Zebrafish (slc25a36-A)/Chicken/Mouse/Human conserved CIS (Table S19), which is not transcribed in any of the variants listed in Table S1, although the whole Mouse sequence is an ORF with frame + 1.

Unless exonized, the conserved purely intronic sequences seem to have no preferred position neither in the genes nor in the individual harboring introns. Rather, as can be expected in case of a random distribution, very large introns may accommodate more than one conserved sequence; for example, the very long first intron of SLC25A21 accommodates three Zebrafish-Chicken-Mouse-Human sequences (Tables S6–S8), while the more “standard” seventh intron of SLC25A21 and fourth intron of SLC25A36 harbor one such sequence each (Tables S9 and S19, respectively).

### 2.9. Percentages of Intronic Conservation

In the carriers studied, the total pool of intronic sequences is 472,638 nucleotides in Zebrafish and 467,846 nucleotides in Chicken.

Table 1 summarizes the total amount (in nucleotides) of Zebrafish intronic sequences which are conserved in Chicken, Mouse and Human and the corresponding percentage over the total number of Zebrafish intronic nucleotides.

Table 2 summarizes the total amount (in nucleotides) of Chicken intronic sequences which are conserved in Mouse and Human and the corresponding percentage over the total number of Chicken intronic nucleotides.

## 3. Discussion

The present results show that in some of the SLC25As genes significant intronic sequences are conserved in Zebrafish, Chicken, Mouse and Human. The last common ancestor of all four species studied is the Euteleostomi (“bony vertebrates”) group which split up about 400 MYA (million years ago) into the Actinopterygii and Sarcopterygii groups. From Sarcopterygii (lobe-finned fishes) derived the extant Birds and Mammals, which in turn mutually diverged some 300–310 MYA. Rodents and Primates diverged quite recently, some 100 MYA only [5,6,7,8,9]. 

The percentages of intronic conservation of Zebrafish sequences in Chicken, Mouse and Human are relatively low and vary somewhat according to the species, between 0.23% and 0.27% (Table 1). The percentages of intronic conservation of Chicken sequences in Mouse and Human are, as expected, much higher (Table 2). It is remarkable that the human conservation (4.40%) is significantly (*p* < 0.01) higher than the mouse conservation (3.20%), by about 37%. For comparison, the Mouse/Human *intronic conservation* averages about 15%, while the *exon conservation* in the mitochondrial carriers of all Vertebrates is about 70%. [10]. The biological nature of the conserved intronic sequences may differ in different genes. The structural arrangement in SLC25A3 is peculiar (Section 2.1). A DNA segment, coding for the first transmembrane segment and parts of the immediately adjoining cytoplasmic and mitochondrial matrix segments of the protein, is duplicated in Zebrafish, Chicken, Mouse and Human. The two copies are located in the interval between exon 1 and exon 3. The splicing machinery is tuned in such a way that only one copy at a time is expressed in these species, forming the second exon of the mRNA transcript. Thus, Zebrafish, Chicken, Mouse and Human express two very similar transcript variants. Contrary to most transcript variants in which different nucleotide sequences are added to or subtracted from the default mRNA product, in SLC25A3 the alternation is between two very similar copies of the exon 2. The unusual structure of the Vertebrate SLC25A3 gene is discussed elsewhere in more detail [4].

Most of the other SLC25A* Zebrafish, Chicken, Mouse or Human CISs are never transcribed, that is they are probably embedded in introns signaled by strong splicing signals. This is the case of another CIS of SLC25A3 (Section 2.1) and all the CISs of SLC25A12 (Section 2.2), SLC25A13 (Section 2.3), SLC25A21 (Section 2.4), SLC25A26 (Section 2.6), and SLC25A36 (Section 2.8).

In SLC25A29 (Section 2.7) there is only one Chicken/Human CIS, harboring stop codons in any frame, both in Chicken and Human. This sequence is never expressed in Chicken, but is expressed in one of the variants of the Human transcripts, the encoded protein being thus truncated and devoid of *Mito_carr* domains.

Some of the Chicken/Human SLC25A21 CISs are present with minor changes in the SLC25A21 genes of other Avian or Mammalian species. In a few instances these changes allow the skipping of the stop codons present in the Chicken and Human genes and such sequences are expressed in normal protein products. In Section 2.4 some transcripts of mallard, white-throated sparrow, gorilla and sperm whale are illustrated, expressing some intronic sequences that are not expressed in Chicken or Human.

Unlike the other genes harboring CISs, the SLC25A25 genes of Zebrafish, Chicken, Mouse and Human harbor two sequences which are expressed in some of the transcripts (Section 2.5 and Table S17). Among the 24 transcripts listed in Table S11, while ten of them do not express any of these sequences, five do express the longer (upstream) sequence but not the shorter (downstream) sequence, seven express the downstream sequence only and two express both sequences. In these sequences the position of the start codon (and first exon) along the gene is very variable and some sort of hierarchical succession seems to operate in the up- to downstream direction: in six variants transcription starts at a first available exon in the gene and the downstream available exon as well as the further downstream long conserved sequence are both skipped; in nine variants the first available exon is skipped and the second available exon is expressed, while the long conserved sequence is skipped; lastly, in seven transcripts in which both upstream available exons are skipped, the start codon is provided by the long conserved sequence. However, in the Chicken transcripts expressing the long conserved sequence as first exon, the sequence is preceded by and fused with an expressed 117-nt sequence. In two very reduced Human transcripts (X3 and X4; Table S17) all three start sequences are skipped and a subsequent, usually expressed, exon is also skipped and transcription starts at the following exon; these transcripts still exhibit the three canonical *Mito_carr* domains, but lack an Ca^2+^ binding site. There seems to be no correlation between the expression of the longer conserved segment and the shorter conserved segment.

Even very long intronic sequences may be conserved in evolutionary distant species, e.g., a 600-bp long stretch in Chicken and Human gluR-C gene [11] or a 2-kb sequence in Birds and Mammals in the PTBP2 gene [12]. In such instances it has been speculated that these intronic stretches may have important regulatory functions, such as regulation of gene expression, and in specific cases this issue has also been addressed experimentally [13,14,15,16,17,18]. Although it would be tempting to hypothesize some specific functional role for the SLC25 CISs which have been conserved over a period as long as 400–450 million years, at present no experimental element supports this view and conservation may also be explained by a random survival in a general scenario of extensive intron re-editing.

## 4. Material and Methods

Genomic Zebrafish (*Danio rerio*), Chicken (*Gallus gallus*), Mouse (*Mus musculus*) and Human (*Homo sapiens*) mitochondrial solute carrier gene (SLC25s genes; members A1 to A54) sequences were retrieved from the NCBI GenBank (http://www.ncbi.nlm.nih.gov/).

Firstly we selected for this study the splice variants of these genes which showed superimposable exon-intron arrangements in all species (mainly from HomoloGene: http://www.ncbi.nlm.nih.gov/homologene/) and assumed these as the “reference” transcripts. Next, we examined, for all the genes showing evidence of Zebrafish/Chicken/Mouse/Human or Chicken/Mouse/Human CISs, all the other transcript variants in order to check whether the CISs present in the “reference” transcripts always held the same intronic position or were, at least in part, exonized in some transcripts.

Alignments of corresponding introns were made by the NCBI BLAST tool (http://blast.ncbi.nlm.nih.gov/Blast.cgi: Align two or more sequences; bl2seq) [19], excluding the low-complexity sequences. Conservation of sequence segments was assumed to be significant only when alignments were Plus/Plus with Expect value 9e-10 or lower and were present in corresponding introns and, when applicable, in the same order in the individual introns. The Multalin (http://multalin.toulouse.inra.fr/multalin/) and MAFFT (http://www.ebi.ac.uk/Tools/msa/mafft/) programs [20] were used to display the alignments between selected intronic sections. Possible exonizations of CISs were sought using the blastx tool (Search protein database using a translated nucleotide query) of NCBI BLAST.

## 5. Conclusions

To conclude, the biological significance of the intron conserved sequences in the SLC25 genes remains unclear. As in other gene families intronic sequences of Euteleostomi have been transferred (through Sarcopterygii) to Birds and (through Sarcopterygii and ancestral Mammals) to Mouse and Humans. A more significant quota of intronic sequences which had emerged anew during Sarcopterygii evolution has been passed to ancestral Mammals and eventually to Mouse and, in a somewhat higher proportion, to Human (Section 2.9, Table 1 and Table 2). Thus, it appears that the evolutionary step from Euteleostomi to Sarcopterygii has been particularly steep, whereas the evolution within the Sarcopterygii group has been relatively more conservative. Furthermore, in a separate investigation we were able to demonstrate that the Chicken and Human intronic sequences largely retain the characteristics of the bird/mammal ancestor sequence, while the Mouse intronic sequences are more divergent, being comparatively richer in C and G and poorer in A and T [21].

Some of the conserved sequences seem to be always “untranscriptable” in every context, even if these are ORFs. Other conserved “intronic” sequences may remain intronic in some transcripts but may become exonized in other transcripts, as in the SLC25A25 of Zebrafish, Chicken, Mouse and Human, or may become transcriptable in other cognate species as a consequence of limited nucleotide mutations, as in the case of SLC25A21. In some instances the expression of DNA sequences which are usually only intronic poses a “threat” to the protein integrity when the stop codons contained in the insert happen to be read in-frame, as in the case of the truncated variant X10 of the Human SLC25A29 (Section 2.7 and Table S18). However, in most cases the expression of “intronic” DNA segments merely inserts novel nucleotide sequences or even novel exons (e.g., the short conserved intronic sequence of SLC25A25; Section 2.5) which do not apparently alter the general structure of the protein product.

Minding the hypothesis of a biological/functional role of the intronic sequences which have been conserved during vertebrate evolution, it seems worth investigating the intronic conservation in other well conserved gene families, for example, the one coding for the “Mitochondrial ribosomal proteins, MRPL1 to MRPL57”.

There is scanty information about possible subtle functional differences between variants expressing “intronic” sequences and those that do not. In this connection, is of special interest the gene SLC25A3. As stated above (Section 2.1), this gene codes for two very similar isoforms, however, the two isoforms strongly differ in their kinetic parameters and furthermore, at least in Ox and Human, one the two isoforms is expressed preferentially in heart and skeletal muscles, whereas the other was equally expressed in all tissues examined [22].

## Figures and Tables

**Table 1:** Intronic Zebrafish nucleotides conserved in Chicken, Mouse and Human.

	Chicken	Mouse	Human
Number of nucleotides	1287	1106	1209
Percentage	0.273	0.234	0.254

**Table 2:** Intronic Chicken nucleotides conserved in Mouse and Human.

	Mouse	Human
Number of nucleotides	14,987	20,601
Percentage	3.203	4.403

## Supplementary Materials

The following are available online at http://www.mdpi.com/2311-553X/5/1/4/s1.
