# Supplementary material for: Conservation of Intronic Sequences in Vertebrate Mitochondrial Solute Carrier Genes (Zebrafish, Chicken, Mouse and Human)
Source: Noncoding RNA. 2019 Jan 6;5(1):4. doi: 10.3390/ncrna5010004 (PMC6468709; doi:10.3390/ncrna5010004)
Supplement: Supplementary file 1 [file ncrna-05-00004-s001.pdf]

Conservation of intronic sequences in Vertebrate mitochondrial solute carrier genes (Zebrafish, Chicken, Mouse and Human)

SUPPLEMENTARY MATERIAL

Sup-Table 1 – The genes considered in this study, with the transcript and protein variants.

		Chrom.	Note	mRNA	Protein
**SLC25A3**	ZEBRAFISH (A3b)	25		NM_213722.1	NP_998887.1
			variant X1	XM_005170654.1	XP_005170711.1
	CHICKEN	1		NM_001006236.1	NP_001006236.1
			variant X1	XM_015284675.1	XP_015140161.1
			variant X2	XM_015284680.1	XP_015140166.1
	MOUSE	10		NM_133668.3	NP_598429.1
			variant X1	XM_006513371.2	XP_006513434.1
	HUMAN	12		NM_005888.3	NP_005879.1
			variant 2	NM_002635.3	NP_002626.1
**SLC25A12**	CHICKEN	7		XM_428938.4	XP_428938.4
	MOUSE	2		NM_172436.	NP_766024.1
			variant X1	XM_011239853.1	XP_011238154.1
			variant X2	XM_011239853.1	XP_011238155.1
			variant X3	XM_006500420.1	XP_006500483.1
	HUMAN	2	variant 1	NM_003705.4	NP_003696.2
			variant X1	XM_005246923.3	XP_005246980.1
			variant X2	XM_011512069.1	XP_011510371.1
			variant X3	XM_011512070.1	XP_011510372.1
**SLC25A13**	CHICKEN	2		NM_001012949.1	NP_001012967.1
	MOUSE	6	variant 1	NM_015829.3	NP_056644.1
			variant 2	NM_001177572.1	NP_001171043.1
			variant X1	XM_006505136.2	XP_006505199.1
			variant X2	XM_006505137.2	XP_006505200.1
	HUMAN	7	variant 1	NM_001160210.1	NP_001153682.1
			variant 2	NM_014251.2	NP_055066.1
			variant X1	XM_006715831.2	XP_006715894.1
			variant X2	XM_011515727.1	XP_011514029.1
			variant X3	XM_011515728.1	XP_011514030.1

**SLC25A21**	ZEBRAFISH	17		NM_001076632.1	NP_001070100.1
			variant X1	XM_005158852.2	XP_005158909.1
			variant X2	XM_009293211.1	XP_009291486.1
	CHICKEN	5	variant X1	XM_001233199.3	XP_001233200.2
			variant X2	XM_421247.4	XP_421247.2
			variant X3	XM_004941769.1	XP_004941826.1
			variant X4	XM_004941770.1	XP_004941827.1
	MOUSE	12	variant 1	NM_172577.3	NP_766165.2
			variant 2	NM_001167976.1	NP_001161448.1
	HUMAN	14	variant 1	NM_030631.3	NP_085134.1
			variant 2	NM_001171170.1	NP_001164641.1
			variant X1	XM_011537287.1	XP_011535589.1
			variant X2	XM_011537288.1	XP_011535590.1
			variant X3	XM_011537289.1	XP_011535591.1
**SLC25A25**	ZEBRAFISH (A25a)	8		NM_213257.1	NP_998422.1
	CHICKEN	17	variant X1	XM_001233164.3	XP_001233165.1
			variant X4	XM_004945811.1	XP_004945868.1
			variant X5	XM_004945812.1	XP_004945869.1
			variant X7	XM_004945814.1	XP_004945871.1
			variant X2	XM_415513.4	XP_415513.3
			variant X6	XM_004945813.1	XP_004945870.1
			variant X3	XM_003642257.2	XP_003642305.1
	MOUSE	2	variant 2	NM_001164357.1	NP_001157829.1
			variant X1	XM_006497954.1	XP_006498017.1
			variant X4	XM_006497958.2	XP_006498021.1
			variant 4	NM_001290558.1	NP_001277487.1
			variant X2	XM_006497955.2	XP_006498018.1
			variant X3	XM_006497957.2	XP_006498020.1
			variant 3	NM_001164358.1	NP_001157830.1
			variant 1	NM_146118.3	NP_666230.2
	HUMAN	9	variant 2	NM_001006641.3	NP_001006642.1
			variant 3	NM_001006642.3	NP_001006643.1
			variant X4	XM_005251689.3	XP_005251746.1
			variant 1	NM_052901.4	NP_443133.2
			variant X2	XM_006716948.1	XP_006717011.1
			variant 5	NM_001265614.2	NP_001252543.1
			variant X3	XM_006716949.2	XP_006717012.1
			variant X1	XM_005251688.1	XP_005251745.1
**SLC25A26**	ZEBRAFISH	11		NM_001030143.1	NP_001025314.1
	CHICKEN	12		NM_001277843.1	NP_001264772.1
	MOUSE	6		NM_026255.5	NP_080531.2

			variant X1	XM_006506517.2	XP_006506580.1
			variant X2	XM_006506518.2	XP_006506581.1
			variant X3	XM_006506519.2	XP_006506582.1
			variant X7	XM_006506520.2	XP_006506583.1
			variant X8	XM_006506522.2	XP_006506585.1
			variant X10	XM_011241446.1	XP_011239748.1
			variant X11	XM_006506523.2	XP_006506586.1
			variant X12	XM_006506524.2	XP_006506587.1
	HUMAN	3	variant 1	NM_173471.3	NP_775742.4
			variant 2	NM_001164796.1	NP_001158268.1
			variant X2	XM_006712956.1	XP_006713019.1
			variant X3	XM_011533327.1	XP_011531629.1
			variant X5	XM_006712957.2	XP_006713020.1
			variant X6	XM_011533328.1	XP_011531630.1
**SLC25A29**	CHICKEN	5	variant X4	XM_421366.3	XP_421366.1
	HUMAN	14	variant 1	NM_001039355.2	NP_001034444.1
			variant 2	NM_001291813.1	NP_001278742.1
			variant 3	NM_001291814.1	NP_001278743.1
			variant 4	NM_152333.3	NP_689546.1
			variant X1	XM_006720038.1	XP_006720101.1
			variant X3	XM_011536443.1	XP_011534745.1
			variant X4	XM_006720039.2	XP_006720102.1
			variant X5	XM_011536444.1	XP_011534746.1
			variant X6	XM_011536445.1	XP_011534747.1
			variant X7	XM_011536446.1	XP_011534748.1
			variant X8	XM_011536447.1	XP_011534749.1
			variant X9	XM_011536448.1	XP_011534750.1
			variant X10	XM_011536449.1	XP_011534751.1
**SLC25A36**	ZEBRAFISH (A36a)	2		NM_001002667.1	NP_001002667.1
			variant X1	XM_005171288.2	XP_005171345.1
			variant X2	XM_009298709.1	XP_009296984.1
			variant X3	XM_009298710.1	XP_009296985.1
	CHICKEN	9		NM_001007960.1	NP_001007961.1
	MOUSE	9		NM_138756.4	NP_620095.1
			variant X1	XM_006510870.2	XP_006510933.1
			variant X2	XM_011242678.1	XP_011240980.1
			variant X8	XM_006510871.2	XP_006510934.1
	HUMAN	3	variant 1	NM_001104647.1	NP_001098117.1
			variant 2	NM_018155.2	NP_060625.2
			variant X1	XM_011512951.1	XP_011511253.1
			variant X2	XM_005247575.3	XP_005247632.1

	variant X3	XM_006713685.2	XP_006713748.1
	variant X4	XM_011512952.1	XP_011511254.1
	variant X5	XM_011512953.1	XP_011511255.1

Sup-Table 2 - SLC25A3. Upstream and downstream conserved DNA sequences of Zebrafish (SLC25A3b), Chicken, Mouse and Human and the corresponding alignments. Consensus line: capital red type = 4/4 identities; normal blue type = 3/4 identities; dot = ≤ 2/4 identities.

UPSTREAM CONSERVED SEQUENCE

```
>ZEBRAFISH (3282-3400)
AGCTGTGAGTTCGGCTCACAGAAATACTTCGTCCTCTGCGGCTTCGGAGGAATCCTGAGCTGCGGCACCACACACACGGCTGTGG
TGCCGCTCGACCTGGTCAAGTGCCGGTTGCAGGT
>CHICKEN (2332-2450)
AGCTGTGAGTATGGCTCGGGCAGATTCTTTATGCTCTGTGGTTTCGGTGGGATTATTAGCTGTGGAACAACACATACAGCACTGG
TTCCTCTAGACCTGGTTAAATGCAGAATGCAGGT
>MOUSE (1380-1498)
AGCTGTGAATATGGATCTGGCAGATTCTTTCTCCTCTGTGGCCTCGGAGGAATTATTAGCTGTGGCACAACACATACAGCATTGG
TTCCTCTAGATCTCATTAAATGCAGAATGCAGGT
>HUMAN (1463-1581)
AGCTGTGACTATGGATCTGGCAGATTCTTTATCCTTTGTGGACTTGGAGGAATTATTAGCTGTGGCACAACACATACAGCATTGG
TTCCTCTAGATCTGGTTAAATGCAGAATGCAGGT
```

```
           1                                                          60
ZEBRAFISH  AGCTGTGAGTTCGGCTCACAGAAATACTTCGTCCTCTGCGGCTTCGGAGGAATCCTGAGC
  CHICKEN  AGCTGTGAGTATGGCTCGGGCAGATTCTTTATGCTCTGTGGTTTCGGTGGGATTATTAGC
    MOUSE  AGCTGTGAATATGGATCTGGCAGATTCTTTCTCCTCTGTGGCCTCGGAGGAATTATTAGC
    HUMAN  AGCTGTGACTATGGATCTGGCAGATTCTTTATCCTTTGTGGACTTGGAGGAATTATTAGC
Consensus  AGCTGTGA.TatGG.TC.ggcAgATtCTTt.TcCTcTGtGG..TcGGaGGaATtaTtAGC

           61                                                       119
ZEBRAFISH  TGCGGCACCACACACACGGCTGTGGTGCCGCTCGACCTGGTCAAGTGCCGGTTGCAGGT
  CHICKEN  TGTGGAACAACACATACAGCACTGGTTCCTCTAGACCTGGTTAAATGCAGAATGCAGGT
    MOUSE  TGTGGCACAACACATACAGCATTGGTTCCTCTAGATCTCATTAAATGCAGAATGCAGGT
    HUMAN  TGTGGCACAACACATACAGCATTGGTTCCTCTAGATCTGGTTAAATGCAGAATGCAGGT
Consensus  TGtGGcACaACACAtACaGCa.TGGTtCCtCTaGA.CTggTtAAaTGCaGaaTGCAGGT
```

Percent Identity Matrix

	ZEBRAFISH	CHICKEN	MOUSE	HUMAN
ZEBRAFISH	-----	72.27	71.43	70.59
CHICKEN	72.27	-----	88.24	89.08
MOUSE	71.43	88.24	-----	94.12
HUMAN	70.59	89.08	94.12	-----

DOWNSTREAM CONSERVED SEQUENCE

```
>ZEBRAFISH (5145-5270)
ACAGCTGTGCGTTCGGCTCAGGGAAATACTACGCTCTGTGCGGGTTCGGGGGCATCCTGAGCTGTGGCATTACACACACGGCCGT
CGTGCCGCTCGATTTGGTGAAGTGCCGCCTGCAGGTCTGTA
>CHICKEN (2609-2734)
ACAGTTGTGAATATGGCTCGCTCAAGTTTTATGCTCTCTGTGGCGTTGGTGGGGTCCTAAGTTGTGGCCTGACACACACTGCTGT
CGTACCTCTGGATTTAGTGAAATGTCGTATGCAGGTTTGTA
>MOUSE (1663-1788)
ACAGTTGTGAATTTGGCTCCATGAAGTATTATGCACTGTGTGGCTTTGGTGGGGTCTTAAGTTGTGGGCTGACACACACTGCTGT
TGTTCCCCTGGACTTAGTAAAGTGCCGCATGCAGGTTTGTA
>HUMAN (1752-1877)
ACAGTTGTGAATTTGGCTCCGCGAAGTATTATGCACTGTGTGGCTTTGGTGGGGTCTTAAGTTGTGGTCTGACACACACTGCTGT
GGTTCCCCTGGATTTAGTGAAATGCCGTATGCAGGTTTGTA
```

```
           1                                                          60
ZEBRAFISH  ACAGCTGTGCGTTCGGCTCAGGGAAATACTACGCTCTGTGCGGGTTCGGGGGCATCCTGA
  CHICKEN  ACAGTTGTGAATATGGCTCGCTCAAGTTTTATGCTCTCTGTGGCGTTGGTGGGGTCCTAA
```

```
MOUSE      ACAGTTGTGAATTTGGCTCCATGAAGTATTATGCACTGTGTGGCTTTGGTGGGGTCTTAA
HUMAN      ACAGTTGTGAATTTGGCTCCGCGAAGTATTATGCACTGTGTGGCTTTGGTGGGGTCTTAA
Consensus  ACAGtTGTGaaTttGGCTC...gAAgTatTAtGC.CTgTGtGGctTtGGtGGggTC.TaA

           61                                                       120
ZEBRAFISH  GCTGTGGCATTACACACACGGCCGTCGTGCCGCTCGATTTGGTGAAGTGCCGCCTGCAGG
CHICKEN    GTTGTGGCCTGACACACACTGCTGTCGTACCTCTGGATTTAGTGAAATGTCGTATGCAGG
MOUSE      GTTGTGGGCTGACACACACTGCTGTTGTTCCCCTGGACTTAGTAAAGTGCCGCATGCAGG
HUMAN      GTTGTGGTCTGACACACACTGCTGTGGTTCCCCTGGATTTAGTGAAATGCCGTATGCAGG
Consensus  GtTGTGG.cTgACACACACtGCtGT.GT.CC.CTgGAtTTaGTgAA.TGcCG.aTGCAGG

           121
ZEBRAFISH  TCTGTA
CHICKEN    TTTGTA
MOUSE      TTTGTA
HUMAN      TTTGTA
Consensus  TtTGTA
```

Percent Identity Matrix

	ZEBRAFISH	CHICKEN	MOUSE	HUMAN
ZEBRAFISH	-----	71.43	73.02	73.81
CHICKEN	71.43	-----	85.71	88.10
MOUSE	73.02	85.71	-----	93.65
HUMAN	73.81	88.10	93.65	-----

Sup-Table 3 - SLC25A3. CIS of Chicken, Mouse and Human and the corresponding alignments. Consensus line: capital red type = 3/3 identities; normal blue type = 2/3 identities; dot = less than two identities.

```
>CHICKEN
ACATGCTCCTTAGATCCATCTTTGTGAGTATCTTTGTGCTGAGTTCTGCTGGATAACTTCAGTTTCTAGCAGTTCCAGTCAAAGT
TGCTCGGCAACTTTTTTGGTTGTACGGTCAAAGATGCTTGAGTCATTGGCATGTGAGAGTAATATAACTGCATGTTAGCTTTCTG
TTCTGATAAAAGTGACTGTAAATATGAGAGTCATTTACCGCTATCAGGACATGGCTGGTATGCAGACATT
>MOUSE
ACATGCTTCCTTAGATCCACCTTTGTGGATGGGTCTTGGACTGAGTTCTACCTGTAAACTTCTTGTTTCTTGTGGTTCCAGTCCA
AGAAACGTCCAGCAACTTTGTTGGTTGTATAGTCGAAGGTGCTTGAGTCATTGGCATGTGAGACTAATATACCTGCATGCTAGTC
TTCAGGTTCTGATGGAAGTGAC
>HUMAN
ACATGCTTCCTTAGATCCACCTTTGTGGATGAATCTTGAACTGAGTTCCACTTGTAAACTTCTTGTTTCTTGTGGTTCCAGTAGT
CAAAGAAACATCCAGCAACTTTTTTGGTTGTATAGTCAAAGGTGCTTGAGTCATTGGCATGTAAGAGAAATATACCTGCATGTTA
GTCTAACGTTCTGATAGAAATGACATGCATTTATGCTGCCATTTGTTACTATCAGGACTCGACTCGTGTGCGGACATT
```

```
           1                                                          60
  CHICKEN  ACATGCT-CCTTAGATCCATCTTTGTGAGTATCT-TTGTGCTGAGTTCTGCTGGATAACT
    MOUSE  ACATGCTTCCTTAGATCCACCTTTGTGGATGGGTCTTGGACTGAGTTCTACCTGTAAACT
    HUMAN  ACATGCTTCCTTAGATCCACCTTTGTGGATGAATCTTGAACTGAGTTCCACTTGTAAACT
Consensus  ACATGCTtCCTTAGATCCAcCTTTGTGgaTg..TcTTG.aCTGAGTTCtaCttGtaAACT

           61                                                        120
  CHICKEN  TCA-GTTTCTAGCAGTTCCAGT---CAAAGTTGCT--CGGCAACTTTTTTGGTTGTACGG
    MOUSE  TCTTGTTTCTTGTGGTTCCAGT---CCAAGAAACGTCCAGCAACTTTGTTGGTTGTATAG
    HUMAN  TCTTGTTTCTTGTGGTTCCAGTAGTCAAAGAAACATCCAGCAACTTTTTTGGTTGTATAG
Consensus  TCttGTTTCTTGtgGTTCCAGT...CaAAGaaaC.tcCaGCAACTTTtTTGGTTGTAtaG

           121                                                       180
  CHICKEN  TCAAAGATGCTTGAGTCATTGGCATGTGAGAGTAATATAACTGCATGTTAGCTTTCTG-T
    MOUSE  TCGAAGGTGCTTGAGTCATTGGCATGTGAGACTAATATACCTGCATGCTAGTCTTCAGGT
    HUMAN  TCAAAGGTGCTTGAGTCATTGGCATGTAAGAGAAATATACCTGCATGTTAGTCTAACG-T
Consensus  TCaAAGgTGCTTGAGTCATTGGCATGTgAGAgtAATATAcCTGCATGtTAGtcTtc.G.T

           181                                                       240
  CHICKEN  TCTGATAAAAGTGAC-TGTAAATATGAGAGTCATTTACCGCTATCAGGACATGGCTGGTA
    MOUSE  TCTGATGGAAGTGAC
    HUMAN  TCTGATAGAAATGACATGCATTTATGC-TGCCATTTGTTACTATCAGGACTCGACTCGTG
Consensus  TCTGATagAAgTGAC.tg.a..tatg...g.cattt....ctatcaggac..g.ct.gt.

           241    250
  CHICKEN  TGCAGACATT
    MOUSE
    HUMAN  TGCGGACATT
Consensus  tgc.gacatt
```

Sup-Table 4 - SLC25A12. CIS of Chicken, Mouse and Human and the corresponding alignments. Consensus line: capital red type = 3/3 identities; normal blue type = 2/3 identities; dot = less than two identities.

```
>CHICKEN
TCTTACGTTTATTTTGAATCTCCGTTATCTCTATTGTTATCTTTGCAGCAGGATATTCTCTTTGTCTTAATAATTTGATGGCAGT
GAAACAGTGGTGTAGTTTAGTAGGATTCAGATTTTTAAGAAGTTAGATGGTGGGATTTGTCAATAACAAATTCCACTTTaaaaaa
aGCCCGTACTGTACTTCAGCAGAGTGCCAGTCAACAGATTCCTTCCCTGCAGGTACACTTACTACCATCATTACAGATCAGATGT
AATGGAGCAGAAGCTATTAGTTTTCTTAAACAAGGAATAGTATTTCTTGCTGCATCTTCTGCCAGCACAAGGGGTAATACGTAAA
TTCAGC
>MOUSE
TTTGGCAGAATTCAAGTCTTTAAGAAGTTAAGTGGGAGGGTTTGTCAATAATGAATTCCATTAAAAAACAACAACAGTTACTGTA
CTTTCACAAAACGCCAGTCAACAGATTTCTCCCCTGCCGATATACTTACCACCGTCATTACAGATCAGATGTAATGGAGCAGAAG
CCAGTATTTTTCTTTAACTAGGAGAGTATTTCTT
>HUMAN
TCTTATGTTTATTTTCGGTCTGATTATCTCTATTGTTATCTTCCCAGTGGGAGCTTTTGTCTTAATAATTTGACCACAGTAAAAC
AGTAGTAGAGTTTGGTAGGATTCAGGTCTTTAAGAAGTTAAGTGGTAGGATTTGTCAATAATGAATTCCATTAAAAAAAAAAAAA
ACAAACCAACAACAGAGTGTACTGTACTTTAACAAAATGCCAGTCAAAAGATTCCTTCCCTGCGGGTATACTTACTCCCATCATT
ACAGATCAGATGTAATGGAGCAGAAGCTATTATTTTTCTTAAACAAGGAAGAATATTTCTTTCCATTCCTTTGCCTGGTATAAGG
GGAATACGTAAATTTAGC
```

```
             1                                                           60
  CHICKEN    TCTTACGTTTATTTTGAATCTCCGTTATCTCTATTGTTATCTTTGCAGCAGGATATTCTC
    MOUSE
    HUMAN    TCTTATGTTTATTTTCGGTCTGA-TTATCTCTATTGTTATCTTCCCAGTGGGAGCTT---
Consensus    tctta.gtttatttt...tct...ttatctctattgttatctt..cag..gga..tt...

             61                                                         120
  CHICKEN    TTTGTCTTAATAATTTGATGGCAGTGAAACAGTGGTGTAGTTTAGTAGGATTCAGATTTT
    MOUSE                                              TTTGGCAGAATTCAAGTCTT
    HUMAN    -TTGTCTTAATAATTTGACCACAGTAAAACAGTAGTAGAGTTTGGTAGGATTCAGGTCTT
Consensus    .ttgtcttaataatttga...cagt.aaacagt.gt..agTTTgGtAGgATTCAggTcTT

             121                                                        180
  CHICKEN    TAAGAAGTTAGATGGTGGGATTTGTCAATAACAAATTCCACTTTAAAAAAAGC-------
    MOUSE    TAAGAAGTTAAGTGGGAGGGTTTGTCAATAATGAATTCCATTAAAAAACAACAA------
    HUMAN    TAAGAAGTTAAGTGGTAGGATTTGTCAATAATGAATTCCATTAAAAAAAAAAAAACAAA
Consensus    TAAGAAGTTAagTGGtaGGaTTTGTCAATAAtgAATTCCAtTaaAAAAaAA.aa......

             181                                                        240
  CHICKEN    CC-----------GTACTGTACTTCAGCAGAGTGCCAGTCAACAGATTCCTTCCCTGCAG
    MOUSE    CAG----------TTACTGTACTTTCACAAAACGCCAGTCAACAGATTTCTCCCCTGCCG
    HUMAN    CCAACAACAGAGTGTACTGTACTTTAACAAAATGCCAGTCAAAAGATTCCTTCCCTGCGG
Consensus    Cc...........gTACTGTACTTtaaCAaAatGCCAGTCAAcAGATTcCTtCCCTGC.G

             241                                                        300
  CHICKEN    GTACACTTACTACCATCATTACAGATCAGATGTAATGGAGCAGAAGCTATTAGTTTTCTT
    MOUSE    ATATACTTACCACCGTCATTACAGATCAGATGTAATGGAGCAGAAGCCAGTATTTTTCTT
    HUMAN    GTATACTTACTCCCATCATTACAGATCAGATGTAATGGAGCAGAAGCTATTATTTTTCTT
Consensus    gTAtACTTACtaCCaTCATTACAGATCAGATGTAATGGAGCAGAAGCtAtTAtTTTTCTT

             301                                                        360
  CHICKEN    AAACAAGGAATAGTATTTCTTGCTGCATCTTCTGCCAGCACAAGGGGTAATACGTAAATT
    MOUSE    TAACTAGGA-GAGTATTTCTT
    HUMAN    AAACAAGGAAGAATATTTCTTTCCATTCCTT-TGCCTGGTATAAGGGGAATACGTAAATT
Consensus    aAACaAGGAagAgTATTTCTT.c.....ctt.tgcc.g....a.ggg.aatacgtaaatt

             361
```

CHICKEN	CAGC
MOUSE	
HUMAN	TAGC
Consensus	.agc

Sup-Table 5 - SLC25A13. CIS of Chicken, Mouse and Human and the corresponding alignments. Consensus line: capital red type = 3/3 identities; normal blue type = 2/3 identities; dot = less than two identities.

>CHICKEN
GGTAACACAAAAGGCTTTGAAGGGGTCAGTATCACCTGAGCAGCTCCACTCTGCTGTTCGCCCAACCATTGCCAGGCGTTGACTT
TTAAGTCCCTAAGGAGCTCGCTTTGCATCATAATTACTGAGAATTAGTACACATGGCTGACTTTTGCACTTGTAACTAAAGCTGA
TTGTTTCATTGAACAAGTCTTTTTCTGTTTTGTAAACTGTTTTCT
>MOUSE
GTGTCACATGAGCAGTTTCTTGCTTTAGCTCAGCCAAGCATCACTGGGCGTTGACTTTTAAGTCCCTTGGAGCTCAGTGTGCTGC
GTGCGTTATTGCTGAGTGTTAGCACACATGGCTGACTTTTCCCTCACGTCAACCAAAGCTGTCCATTCCTTTGAACAA
>HUMAN
GGGAA*T*ACAAAGGGTTTTGTAAGGTTGTGTCACATGAGCGGTTCCTCATCTTAGTTCATCCAAGCATTGCTAGGT*G*TTGACTTTT
AAGTCCCTGGGGAGCTCACCTTGCTTCATGATTACTGAGAATTAGTACACATGGCTGACT*T*TTGCACTTGTAACCAAAGCTGCTC
ATTTCTTTGAACAAGGCTTATTCTGTTTTATGATCTGTTTTCT

```
           1                                                          60
  CHICKEN  GGTAACACAAAAGGCTTTGAAGGGGTCAGTATCACCTGAGCAGCTCCACTCTGCTGTTCG
    MOUSE                              GTGTCACATGAGCAGTTTCTTGCTTTAGCTCA
    HUMAN  GGGAATACAAAGGGTTTTGTAAGGTT--GTGTCACATGAGCGGTTCCTCATCTTAGTTCA
Consensus  gg.aa.acaaa.gg.tttg.a.gg.t..GTgTCACaTGAGCaGtTcCtc.ctttaGtTCa

           61                                                        120
  CHICKEN  CCCAACCATTGCCAGGCGTTGACTTTTAAGTCCCTAAGGAGCTCGCTTTGCATCATA---
    MOUSE  GCCAAGCATCACTGGGCGTTGACTTTTAAGTCCCTT-GGAGCTCAGTGTGCTGCGTGCGT
    HUMAN  TCCAAGCATTGCTAGGTGTTGACTTTTAAGTCCCTGGGGAGCTCACCTTGCTTCATG---
Consensus  .CCAAgCATtgCtaGGcGTTGACTTTTAAGTCCCT..GGAGCTCacttTGCttCaTg...

           121                                                       180
  CHICKEN  -ATTACTGAGAATTAGTACACATGGCTGACTTTTGCA-CTTGT-AACTAAAGCTGATTGT
    MOUSE  TATTGCTGAGTGTTAGCACACATGGCTGACTTTTCCCTCACGTCAACCAAAGCTGTCCAT
    HUMAN  -ATTACTGAGAATTAGTACACATGGCTGACTTTTGCA-CTTGT-AACCAAAGCTGCTCAT
Consensus  .ATTaCTGAGaaTTAGtACACATGGCTGACTTTTgCa.CttGT.AACcAAAGCTG.tcaT

           181                                     221
  CHICKEN  TTCATTGAACAAGTCTTTTTCTGTTTTGTAAACTGTTTTCT
    MOUSE  TCCTTTGAACAA
    HUMAN  TTCTTTGAACAAGGCTTATTCTGTTTTATGATCTGTTTTCT
Consensus  TtCtTTGAACAAg.ctt.ttctgtttt.t.a.ctgttttct
```

Sup-Table 6 – SCL25A21. CIS of Zebrafish, Chicken, Mouse and Human and the corresponding alignments. Consensus line in 4-sequence alignments: capital red type = 4/4 identities; normal blue type = 3/4 identities; dot = ≤ 2/4 identities. Zebrafish/Mouse consensus at the beginning and Zebrafish/Chicken consensus at the end of the alignments are indicated in normal black type.

```
>ZEBRAFISH
AAACTGTCACCGCATCAGCCGCAAGAGTCTGGGGAGTTGAACTTGACTGGAACATATGTCTTCAATTTTAATAGCCCTTCATTCC
CCCAGATGGCCTGGAGGGGGGAAGGTTGCCTGAGAAGCTGCGTTTCATGTAAGAATAATTTGTGTTAAGTAGCTGGGCGATCTGT
TTGTTACGCTAGAGCTGACAGTTTGTTTATGTTTCTTGAGGAGTATGATGGATGGGGCAGGAGTTGATGCTAATTAGGCTAAATT
GTTGCTAAGAATGCAGTGGGCCTGAGCCACACTGCATAGGCCAGCTCCCATTGTTCCTGTCTCTTCGCACTCGAGGCGTAATTGA
ATGGAGCTTGCAGCCATTTATCAT
>CHICKEN
GGAGTTAAGTTTGACTGAAACATATGTCTTCAATTTTAATAGCTCAGCTTCCCAGATGCCCTAGAGGAGAATATTGCCTGAGAAG
GAGCTTTTCATGTAGGAATAATTTGTGTTAAGTAGCTCAGTGATCTGTTTGTTAGGTTAGAGCTGACAGTTTGTTTATATTTCTA
AAGGGGTATGATGAATGGGGGAGAAGTTGATGCTAATTAGCCTAAATTGTTCCTAAGAATGCAGAGCACTTTGCCAGTTTCATTG
TTTCTGTTTCTTTGCACATTTTCAATAATTGAATGGAGCTCAGACCATTCATCAT
>MOUSE
AAACTGTCACAGCAGCAATCATTACAGTCTGGGAGCCAAGTTTGACTGCAACATATGTCTTCAATTTTAATAGCTCAGTTCCCCA
GATGCCCTAAAGGAGAATATTGCCTGAGAAGGAGCTTTTCATGTAAGAATAATTTGTGTTAAGTAGCTCAGTGATCTGTTTGTTA
GGTTAGAGCTGACAGTTTGTTTATATTTCTAAAGGGGTATGATGAATGGGGGAGAAGTTGATGCTAATTAGCCCAAATTGTTCCT
AAGAATGTAGAGGCCTTTGCCA
>HUMAN
GGAGTTGAGTTTGACTGGAACATATGTCTTCAATTTTAATAGCTCAGCTTCCCAGATGCCCTAAAGGAGAATATTGCCTGAGAAG
GAGCTTTTCATGTAAGAATAATTTGTGTTAAGTAGCTCAGTGATCTGTTTGTTAGGTTACAGCTGACAGTTTGTTTATATTTCTA
AAGGGGTATGATGAATGGGGGAGAAGTTGATGCTAATTAGCCTAAATTGTTCCTAAGAATGTAGAGGG
```

```
            1                                                         60
 ZEBRAFISH  AAACTGTCACCGCATCAGCCGCAAGAGTCTGGGGAGTTGAACTTGACTGGAACATATGTC
   CHICKEN                                  GGAGTTAAGTTTGACTGAAACATATGTC
     MOUSE  AAACTGTCACAGCAGCAATCATTACAGTCTGGG-AGCCAAGTTTGACTGCAACATATGTC
     HUMAN                                  GGAGTTGAGTTTGACTGGAACATATGTC
 Consensus  aaactgtcac.gca.ca..c...a.agtctggGgAGttgAgtTTGACTGgAACATATGTC

            61                                                       120
 ZEBRAFISH  TTCAATTTTAATAGCCCTTCATTCCCCCAGATGGCCTGGAGGGGGGAAGGTTGCCTGAGA
   CHICKEN  TTCAATTTTAATAGCTCAGCTT---CCCAGATGCCCTAGAGGAG--AATATTGCCTGAGA
     MOUSE  TTCAATTTTAATAGCTCAGTT---CCCCAGATGCCCTAAAGGAG--AATATTGCCTGAGA
     HUMAN  TTCAATTTTAATAGCTCAGCTT---CCCAGATGCCCTAAAGGAG--AATATTGCCTGAGA
 Consensus  TTCAATTTTAATAGCtCagctt...CCCAGATGcCCTaaAGGaG..AAtaTTGCCTGAGA

            121                                                      180
 ZEBRAFISH  AGCTGCGTTTCATGTAAGAATAATTTGTGTTAAGTAGCTGGGCGATCTGTTTGTTACGCT
   CHICKEN  AGGAGCTTTTCATGTAGGAATAATTTGTGTTAAGTAGCTCAGTGATCTGTTTGTTAGGTT
     MOUSE  AGGAGCTTTTCATGTAAGAATAATTTGTGTTAAGTAGCTCAGTGATCTGTTTGTTAGGTT
     HUMAN  AGGAGCTTTTCATGTAAGAATAATTTGTGTTAAGTAGCTCAGTGATCTGTTTGTTAGGTT
 Consensus  AGgaGCtTTTCATGTAaGAATAATTTGTGTTAAGTAGCTcaGtGATCTGTTTGTTAgGtT

            181                                                      240
 ZEBRAFISH  AGAGCTGACAGTTTGTTTATGTTTCTTGAGGAGTATGATGGATGGGGCAGGAGTTGATGC
   CHICKEN  AGAGCTGACAGTTTGTTTATATTTCTAAAGGGGTATGATGAATGGGGGAGAAGTTGATGC
     MOUSE  AGAGCTGACAGTTTGTTTATATTTCTAAAGGGGTATGATGAATGGGGGAGAAGTTGATGC
     HUMAN  ACAGCTGACAGTTTGTTTATATTTCTAAAGGGGTATGATGAATGGGGGAGAAGTTGATGC
 Consensus  AgAGCTGACAGTTTGTTTATaTTTCTaaAGGgGTATGATGaATGGGGgAGaAGTTGATGC

            241                                                      300
 ZEBRAFISH  TAATTAGGCTAAATTGTTGCTAAGAATGCAGTGGGCCTGAGCCACACTGCATAGGCCAGC
   CHICKEN  TAATTAGCCTAAATTGTTCCTAAGAATGCAGAG---------CACTTTGC------CAGT
     MOUSE  TAATTAGCCCAAATTGTTCCTAAGAATGTAGAGG---------CCTTTGCCA
```

```
    HUMAN  TAATTAGCCTAAATTGTTCCTAAGAATGTAGAGGG
Consensus  TAATTAGcCtAAATTGTTcCTAAGAATGtAGaGgg.........ctttgc......cagt

           301                                                        360
ZEBRAFISH  TCCCATTGTTCCTGTCTCTTCGCACTCGAGGCGTAATTGAATGGAGCTTGCAGCCATTTA
  CHICKEN  TTC-ATTGTTTCTGTTTCTTTGCACATTTTCAATAATTGAATGGAGCTCAGA-CCATTCA
    MOUSE
    HUMAN
Consensus  t.c.attgtt.ctgt.tctt.gcac........taattgaatggagct...a.ccatt.a

           361
ZEBRAFISH  TCAT
  CHICKEN  TCAT
    MOUSE
    HUMAN
Consensus  tcat
```

Percent Identity Matrix

	ZEBRAFISH	CHICKEN	MOUSE	HUMAN
ZEBRAFISH	-----	81.29	82.67	86.55
CHICKEN	81.29	-----	95.53	96.64
MOUSE	82.67	95.53	-----	96.22
HUMAN	86.55	96.64	96.22	-----

<u>Sup-Table 7</u> - SCL25A21. A CIS of Zebrafish, Chicken, Mouse and Human and the corresponding alignments. Consensus line: capital red type = 4/4 identities; normal blue type = 3/4 identities; dot = ≤ 2/4 identities.

```
>ZEBRAFISH
TAAGCAAAAGCAGATGTTATGAAGTTTACAGAAATCAGAACATTATTTTCCTGTTTCCATTGCATTCTTCTAAACACTTAGTGAA
CAAGCTTTGAAG
>CHICKEN
TAAGTAAAAGCAGATGTTGCAAAGTTTAGAAAATCAAGACATTATTTTCCTGTTTCCACTGAATATTTCTAAACACATTATAAAT
AAGCTTTGAAG
>MOUSE
TAAGTAAAAACAGATGTTTCGAAGTTTTGAAAATCAAGACATTTTTTGTTTCCATTGAATTTTTAAAAGCATTATAAATAAGCTT
TGAAG
>HUMAN
TAAGTAAAAGCAGATGTTGTAAAGTTTAGAAAATCAAGACATTATTTTCCTGTTTCCATTGAATTTTTTCTAAACACATTATAAA
TAAGCTTTGAAG
```

```
            1                                                          60
 ZEBRAFISH  TAAGCAAAAGCAGATGTTATGAAGTTTACAGAAATCAGAACATTATTTTCCTGTTTCCAT
   CHICKEN  TAAGTAAAAGCAGATGTTGCAAAGTTTAGA-AAATCAAGACATTATTTTCCTGTTTCCAC
     MOUSE  TAAGTAAAAACAGATGTTTCGAAGTTTTGA-AAATCAAGACATTTTTT----GTTTCCAT
     HUMAN  TAAGTAAAAGCAGATGTTGTAAAGTTTAGA-AAATCAAGACATTATTTTCCTGTTTCCAT
 Consensus  TAAGtAAAAgCAGATGTT...AAGTTTagA.AAATCAagACATTaTTTtcctGTTTCCAt

            61                                    98
 ZEBRAFISH  TGCATTCTT-CTAAACACTTAGTGAACAAGCTTTGAAG
   CHICKEN  TGAATATTT-CTAAACACATTATAAATAAGCTTTGAAG
     MOUSE  TGAATTTTT---AAAAGCATTATAAATAAGCTTTGAAG
     HUMAN  TGAATTTTTTCTAAACACATTATAAATAAGCTTTGAAG
 Consensus  TGaATttTT.ctAAAcaCaTtaTaAAtAAGCTTTGAAG
```

Percent Identity Matrix

	ZEBRAFISH	CHICKEN	MOUSE	HUMAN
ZEBRAFISH	-----	82.29	76.67	85.42
CHICKEN	82.29	-----	88.89	96.88
MOUSE	76.67	88.89	-----	88.89
HUMAN	85.42	96.88	88.89	-----

<u>Sup-Table 8</u> - SCL25A21. CIS of Zebrafish, Chicken, Mouse and Human and the corresponding alignments. Consensus line: capital red type = 4/4 identities; normal blue type = 3/4 identities; dot = ≤ 2/4 identities.

```
>ZEBRAFISH
GCATAAGCGAATAATTAGGTAATTGCCCTCCTGATTGAGCCCAATAGGAAACGTTTCAATTAGGCTAAAACTGCGCGAGGAGGAA
GTAAGCTCTTACAAAGGGAAACATTTCTTGTCATGCAACTGTCAGCTCCACGAAGCCCACACCAAAAGCAAGGATTTACTGGAT
>CHICKEN
GCATAAGTGAGTAATTAGATAATTGCTGCCTTGATTGAGCTTAATAGGAAACATTTCAATTAGGCTAAAACTACACAGAAAGGAA
GTAGGCTTTTACAAAGGGAAACATTCCATGTCATGCAACTGTCAGCTC
>MOUSE
GCATAAGTGAGTAATTAGATAATTGCTGCCTTGATTGAGCTTAATAGGAAACATTTCAATTAGGCTAAAACTACACAGAAAGGAA
GTGGGTTTTTTTTTACAAAGGGAAACATTCCTTGTCATGCAACTGTCAGCTTGTGCCCACACTAAGAGATGGGATTAAGTGGAT
>HUMAN
GCATAAGTGAGTAATTAGATAATTGCTGCCTTGATTGAGCTTAATAGGAAACATTTCAATTAGGCTAAAACTACACAGAAAGGAA
GTGGGTTTTTTACAAAGGGAAACATTCCTTGTCATGCAACTGTCAGCTTGTGCCCACACTAAGAGATGGGATTAAGTGGAT
```

```
                1                                                          60
 ZEBRAFISH  GCATAAGCGAATAATTAGGTAATTGCCCTCCTGATTGAGCCCAATAGGAAACGTTTCAAT
   CHICKEN  GCATAAGTGAGTAATTAGATAATTGCTGCCTTGATTGAGCTTAATAGGAAACATTTCAAT
     MOUSE  GCATAAGTGAGTAATTAGATAATTGCTGCCTTGATTGAGCTTAATAGGAAACATTTCAAT
     HUMAN  GCATAAGTGAGTAATTAGATAATTGCTGCCTTGATTGAGCTTAATAGGAAACATTTCAAT
 Consensus  GCATAAGtGAgTAATTAGaTAATTGCtgcCtTGATTGAGCttAATAGGAAACaTTTCAAT

                61                                                        120
 ZEBRAFISH  TAGGCTAAAACTGCGCGAGGAGGAAGTAAGCTCTT----ACAAAGGGAAACATTTCTTGT
   CHICKEN  TAGGCTAAAACTACACAGAAAGGAAGTAGGCTTTT----ACAAAGGGAAACATTCCATGT
     MOUSE  TAGGCTAAAACTACACAGAAAGGAAGTGGGTTTTTTTTTACAAAGGGAAACATTCCTTGT
     HUMAN  TAGGCTAAAACTACACAGAAAGGAAGTGGGTTTTTT---ACAAAGGGAAACATTCCTTGT
 Consensus  TAGGCTAAAACTaCaCagaaAGGAAGT.gGcTtTT....ACAAAGGGAAACATTcCtTGT

                121                                                173
 ZEBRAFISH  CATGCAACTGTCAGCTCCACGAAGCCCACACCAAAAGCAAGGATTTACTGGAT
   CHICKEN  CATGCAACTGTCAGCTC
     MOUSE  CATGCAACTGTCAGCTT----GTGCCCACACTAAGAGATGGGATTAAGTGGAT
     HUMAN  CATGCAACTGTCAGCTT----GTGCCCACACTAAGAGATGGGATTAAGTGGAT
 Consensus  CATGCAACTGTCAGCTc......gcccacac.aa.ag...ggatt.a.tggat
```

Percent Identity Matrix

	ZEBRAFISH	CHICKEN	MOUSE	HUMAN
ZEBRAFISH	-----	84.96	81.21	81.21
CHICKEN	84.96	-----	96.99	96.99
MOUSE	81.21	96.99	-----	100.00
HUMAN	81.21	96.99	100.00	-----

<u>Sup-Table 9</u> - SCL25A21. CIS of Zebrafish, Chicken, Mouse and Human and the corresponding alignments. Consensus line: capital red type = 4/4 identities; normal blue type = 3/4 identities; dot = ≤ 2/4 identities.

```
>ZEBRAFISH
CTGTCATTTGTAAAAAATATCCTGGCCAATTAGATGTCTTGGTTCGTGCTTCTTTTAGGTACAGTATTACACCAGTGTCGAGTCG
CAGACCAATTAACCAAATTGTCCCCATATGCCTCCCAGTCTGCCTGCATGAACTCGGTTTATAAATCCT
>CHICKEN
CTGTCATTTGTAAAATATTCTGGCCAATTAGCTAATAGTGTGCTTGGATTTCTCCTGTTTTGATACAGTAATACTAAGTACATTG
TGAAGCCCAATTATACAAATCATCCCCATATGCCATACCAGTCTTTCTTTATGAACTGTGTTTATAAATCCT
>MOUSE
CTGTCATTTGTAAAATATTCTGGCCAATTAGCTAATAGTGTGCTTGGATTTCTCCTGTTTTGATACAGTAATACTAAGTACATTG
TGAAGCCCAATTATACAAATCATCCCCATATGCCGTACCAGTCTTTCTTTATGAACTGTGTTTATAAATCCT
>HUMAN
CTGTCATTTGTAAAATATTCTGGCCAATTAGCTAATAGTGTGCTTGGATTTCTCCTGTTTTGATACAGTAATACTAAGTACATTG
TGAAGCCCAATTATACAAATCATCCCCATATGCCATACCAGTCTTTCTTTATGAACTGTGTTTATAAATCCT
```

```
            1                                                          60
 ZEBRAFISH  CTGTCATTTGTAAAAAATATCCTGGCCAATTAGATGTCTTGGTTCGTG-----CTTCT-T
   CHICKEN  CTGTCATTTGTAAAA--TATTCTGGCCAATTAGCTAATAGTGTGCTTGGATTTCTCCTGT
     MOUSE  CTGTCATTTGTAAAA--TATTCTGGCCAATTAGCTAATAGTGTGCTTGGATTTCTCCTGT
     HUMAN  CTGTCATTTGTAAAA--TATTCTGGCCAATTAGCTAATAGTGTGCTTGGATTTCTCCTGT
 Consensus  CTGTCATTTGTAAAA..TATtCTGGCCAATTAGcTaatagtGTgCtTGgatttCTcCTgT

            61                                                        120
 ZEBRAFISH  TTAGGTACAGTATTACACCAGTGTCGAGTCGCAGACCAATTAACCAAATTGTCCCCATAT
   CHICKEN  TTTGATACAGTAATACTA-AGTACATTGT-GAAGCCCAATTATACAAATCATCCCCATAT
     MOUSE  TTTGATACAGTAATACTA-AGTACATTGT-GAAGCCCAATTATACAAATCATCCCCATAT
     HUMAN  TTTGATACAGTAATACTA-AGTACATTGT-GAAGCCCAATTATACAAATCATCCCCATAT
 Consensus  TTtGaTACAGTAaTACta.AGTacattGT.GaAGcCCAATTAtaCAAATcaTCCCCATAT

            121                                     161
 ZEBRAFISH  GCC-TCCCAGTCTGCCTGCATGAACTCGGTTTATAAATCCT
   CHICKEN  GCCATACCAGTCTTTCTTTATGAACTGTGTTTATAAATCCT
     MOUSE  GCCGTACCAGTCTTTCTTTATGAACTGTGTTTATAAATCCT
     HUMAN  GCCATACCAGTCTTTCTTTATGAACTGTGTTTATAAATCCT
 Consensus  GCC.TaCCAGTCTttCTttATGAACTgtGTTTATAAATCCT
```

Percent Identity Matrix

	ZEBRAFISH	CHICKEN	MOUSE	HUMAN
ZEBRAFISH	-----	75.68	75.68	75.68
CHICKEN	75.68	-----	99.36	100.00
MOUSE	75.68	99.36	-----	100.00
HUMAN	75.68	100.00	100.00	-----

<u>Sup-Table 10</u> - SCL25A21. CIS of Chicken, Mouse and Human and the corresponding alignments. Consensus line: capital red type = 3/3 identities; normal blue type = 2/3 identities; dot = less than two identities.

```
>CHICKEN
TGGCATTTCCTGTTGGTTGAAATTTTGGTTTGCATACTAAGTTAATATTTAATGTTATCATTGGCAAGGCAAACTTCTGTAGGAA
AATATCAGTGTGAATTTTACTAGTGAGATGGGAGTACAGGCCATAAAAATTCTCAGTTTCCCTTTTAAAAACTTAATCCATTTGT
CCAACAGGAATGAATACAGAAGGAGTAGAGGTTTTGAACTTCCCTCACATCTGGGTAAAGGAGTGTATTTTGACTCCAAGCACAA
TAAAACAATCGTACTTGCTGGTGGTATATGACATAATGACCCAAATATGTTTTTAAAAAATCATTTATTGCTGTACATAAACAGC
TAAGTTAGCACAAGGGATGATAAAATAACATTGGTAATTCTTCCTTTAGCTGGGAACCATTGGTGGCCCATTAGGATTCCAGATG
TAGCCATGTGTGCTTTTCCATTTGTTTTCTAATGTCTAGTGCTTGGACCCTTGGTACCTTGGCTGTGGTTGTCAAGCATGTTGGC
TTTTCAGCTTGAGGTCTTCCTGGAGAAACACACTGGCCTGGCTAATAGCTATAAAGCAGCTTATTGTCTTGGGCCCAGTTTGTTG
GGACTAGCTGGAGTGAGGGCAGCTTTATGTCAAAGGTCAAACACCGGCTTGACCCA
>MOUSE
TGGCATTTCCTGTTGCTTGGAAGCTGTTTGGCTTTCTGCATTAGTATTTAGTGTTATCATTGGCCAAGCACCTTTGCTGGGTAGA
AAAATACTGGTGCAAATTTTACTGGTAAGGTGGGAATAGAAGTCATAAAAATTCTCAATTTCCCTTTTAAAAACTTAACTCATTT
GTCCAAGAGGAATGAACACAGAAGGACAAGAGATTTTAAACTTCCCGTACATCTGGGCACAGCCTTGTATTTTGACTCCAAGCAC
AATAAAACAACCTTACTTGCTGGTGGTATATGACATAATGACCCAAATATGTTTTTAAAAAATCATTTATTGCTGTACATAAACA
GCAAAGTTAGCGCAAGGGATGATAAAATAACACGGGTAATTCTTCCCTGTGCTGGGAACCATCGGCGGCCCATTAGGATTCCAGA
TGTAGCTCTCCCCCATTTGTTTTCCAATGTCTAGTGCTTAGACCCTTGGTACCTTGACGGTGGTTGTCAAGCATGTTGGCTTTTC
AGCTTGAGGTCTTCCTTGGAGAAACACACTGATCTGGCCCAGGGCTATAAAGCACACTATTGTCTTGGGCCCAGTTTGTTGAGAA
TATGAGACCCAGGGCAGCCTCCTGCCAAAGGTCAAATACCAGTTTGACCCA
>HUMAN
TGGCATTTCCTGTTGGTTGGAAGATGTTTGGCTTTCTGCATTTGTATTTAGTGTTATCATTGGCCAGGCACCTTTGCTGGGTAGA
AAAATACTGGTGCAAATTTTACTGGCAAGGTGGGAGTAAAAGCCATAAAAATTCTCAATTTTCCTTTTAAAAACTTAACTCATTT
GTCCAAGAGAAATGAATACAGAAGGACAAGAGATTTTTAAACTTCCCGTACATCTGGGTATAGCAGTGTATTTTGACTCCAAGCA
CAATAAAACAACCTTACTTGCTGGTGGTATATGACATAATGACCCAAATATGTTTTTAAAAAATCATTTATTGCTGTACATAAAC
AGCAAAGTTAGCGCAAGGGATGATAAAATAACATGGGTAATTCTTCCTTTAGCTGGGAACCATTGGCGGCCCATTAGGATTCCAG
ATGTAGCTGTGTGTGCTTTTCCATTTGTTTTCCAGTGTCTAGTGCTTATGCCCTTGGTACCTTGATGGTGGTTGTCAGGCATGTT
GGCTTTTCAGCTTGAGGTCTTCCTTGGAGAAACACAGTGATCGGGCTCTGGGCTATAAAGCACACTATTGTCTTGGGCCCAGTTT
GTTGAGAATATAAGACCCAGGGCAGCCTCCTGCCAAAGGTCAAATACCAGCTTGACCCA
```

```
              1                                                          60
  CHICKEN     TGGCATTTCCTGTTGGTTGAAATTTTGGTTTGCATACTAAGTTAATATTTAATGTTATCA
    MOUSE     TGGCATTTCCTGTTGCTTGGAAGCT-GTTTGGCTTTCTGCATTAGTATTTAGTGTTATCA
    HUMAN     TGGCATTTCCTGTTGGTTGGAAGAT-GTTTGGCTTTCTGCATTTGTATTTAGTGTTATCA
Consensus     TGGCATTTCCTGTTGgTTGgAAg.T.GtTTgGCtTtCTgcaTTagTATTTAgTGTTATCA

              61                                                        120
  CHICKEN     TTGGCAAGGCAAACTT-CTG--TAGGAAAATATCAGTGTGAATTTTACTAGTGAGATGGG
    MOUSE     TTGGCCAAGCACCTTTGCTGGGTAGAAAAATACTGGTGCAAATTTTACTGGTAAGGTGGG
    HUMAN     TTGGCCAGGCACCTTTGCTGGGTAGAAAAATACTGGTGCAAATTTTACTGGCAAGGTGGG
Consensus     TTGGCcAgGCAcctTTgCTGggTAGaAAAATActgGTGcaAATTTTACTgGtaAGgTGGG

              121                                                       180
  CHICKEN     AGTACAGGCCATAAAAATTCTCAGTTTCCCTTTTAAAAACTTAATCCATTTGTCCAACAG
    MOUSE     AATAGAAGTCATAAAAATTCTCAATTTCCCTTTTAAAAACTTAACTCATTTGTCCAAGAG
    HUMAN     AGTAAAAGCCATAAAAATTCTCAATTTTCCTTTTAAAAACTTAACTCATTTGTCCAAGAG
Consensus     AgTA.AaGcCATAAAAATTCTCAaTTTcCCTTTTAAAAACTTAActCATTTGTCCAAgAG

              181                                                       240
  CHICKEN     GAATGAATACAGAAGGAGTAGAGGTTTT-GAACTTCCCTCACATCTGGGTAAAGGAGTGT
    MOUSE     GAATGAACACAGAAGGACAAGAGATTTT-AAACTTCCCGTACATCTGGGCACAGCCTTGT
    HUMAN     AAATGAATACAGAAGGACAAGAGATTTTTAAACTTCCCGTACATCTGGGTATAGCAGTGT
Consensus     gAATGAAtACAGAAGGAcaAGAGaTTTT.aAACTTCCCgtACATCTGGGtA.AGcagTGT

              241                                                       300
  CHICKEN     ATTTTGACTCCAAGCACAATAAAACAATCGTACTTGCTGGTGGTATATGACATAATGACC
    MOUSE     ATTTTGACTCCAAGCACAATAAAACAACCTTACTTGCTGGTGGTATATGACATAATGACC
```

```
    HUMAN  ATTTTGACTCCAAGCACAATAAAACAACCTTACTTGCTGGTGGTATATGACATAATGACC
Consensus  ATTTTGACTCCAAGCACAATAAAACAAcCtTACTTGCTGGTGGTATATGACATAATGACC

           301                                                      360
  CHICKEN  CAAATATGTTTTTAAAAAATCATTTATTGCTGTACATAAACAGCTAAGTTAGCACAAGGG
    MOUSE  CAAATATGTTTTTAAAAAATCATTTATTGCTGTACATAAACAGCAAAGTTAGCGCAAGGG
    HUMAN  CAAATATGTTTTTAAAAAATCATTTATTGCTGTACATAAACAGCAAAGTTAGCGCAAGGG
Consensus  CAAATATGTTTTTAAAAAATCATTTATTGCTGTACATAAACAGCaAAGTTAGCgCAAGGG

           361                                                      420
  CHICKEN  ATGATAAAATAACATTGGTAATTCTTCCTTTAGCTGGGAACCATTGGTGGCCCATTAGGA
    MOUSE  ATGATAAAATAACACGGGTAATTCTTCCCTGTGCTGGGAACCATCGGCGGCCCATTAGGA
    HUMAN  ATGATAAAATAACATGGGTAATTCTTCCTTTAGCTGGGAACCATTGGCGGCCCATTAGGA
Consensus  ATGATAAAATAACAtgGGTAATTCTTCCtTtaGCTGGGAACCATtGGcGGCCCATTAGGA

           421                                                      480
  CHICKEN  TTCCAGATGTAGCCATGTGTGCTTTTCCATTTGTTTTCTAATGTCTAGTGCTTGGACCCT
    MOUSE  TTCCAGATGTAGCT-------CTCCCCCATTTGTTTTCCAATGTCTAGTGCTTAGACCCT
    HUMAN  TTCCAGATGTAGCTGTGTGTGCTTTTCCATTTGTTTTCCAGTGTCTAGTGCTTATGCCCT
Consensus  TTCCAGATGTAGCt.tgtgtgCTtttCCATTTGTTTTCcAaTGTCTAGTGCTTagaCCCT

           481                                                      540
  CHICKEN  TGGTACCTTGGCTGTGGTTGTCAAGCATGTTGGCTTTTCAGCTTGAGGTCTTCCT-GGAG
    MOUSE  TGGTACCTTGACGGTGGTTGTCAAGCATGTTGGCTTTTCAGCTTGAGGTCTTCCTTGGAG
    HUMAN  TGGTACCTTGATGGTGGTTGTCAGGCATGTTGGCTTTTCAGCTTGAGGTCTTCCTTGGAG
Consensus  TGGTACCTTGacgGTGGTTGTCAaGCATGTTGGCTTTTCAGCTTGAGGTCTTCCTtGGAG

           541                                                      600
  CHICKEN  AAACACACTGGCCTGGCTAATAGCTATAAAGCAGCTTATTGTCTTGGGCCCAGTTTGTTG
    MOUSE  AAACACACTGATCTGGCCCAGGGCTATAAAGCACACTATTGTCTTGGGCCCAGTTTGTTG
    HUMAN  AAACACAGTGATCGGGCTCTGGGCTATAAAGCACACTATTGTCTTGGGCCCAGTTTGTTG
Consensus  AAACACAcTGatCtGGCtcaggGCTATAAAGCAcacTATTGTCTTGGGCCCAGTTTGTTG

           601                                                656
  CHICKEN  GGACTAGCTGGAGTGAGGGCAGCTTTATGTCAAAGGTCAAACACCGGCTTGACCCA
    MOUSE  AGAATA-TGAGACCCAGGGCAGCCTCCTGCCAAAGGTCAAATACCAGTTTGACCCA
    HUMAN  AGAATA-TAAGACCCAGGGCAGCCTCCTGCCAAAGGTCAAATACCAGCTTGACCCA
Consensus  aGAaTA.t.aGAcccAGGGCAGCcTccTGcCAAAGGTCAAAtACCaGcTTGACCCA
```

Percent Identity Matrix

	CHICKEN	MOUSE	HUMAN
CHICKEN	-----	85.67	86.59
MOUSE	85.67	-----	94.74
HUMAN	86.59	94.74	-----

<u>Sup-Table 11</u> - SCL25A21. CIS of Chicken, Mouse and Human and the corresponding alignments. Consensus line: capital red type = 3/3 identities; normal blue type = 2/3 identities; dot = less than two identities.

```
>CHICKEN
ATACTGTGCCTGTTTAAATCACTAATATATTTTAATTGTTTCCATCTTTCAAAATATATCTAACAAGTTCAATTTTTAGTTGTTG
TTACACAAATAAACTGGTATTAGATGCATTGCTTTCTTTCTTTCAAACATACTTTCAAATTACTGCTTAATGTGGTTTGAAGATT
TTGCCAACATAACCTTCCAGATCCTTTTCTTGTATGAGGGTGGTTGATTACATCTATCTTTTGGTTTACAAATGCCAATTTATTC
CAAAGAATGCAAACCCTGGGACCATTTGTCATTACACTTTGATTGTAGCTCACTTCATTATTGTATGTAGGAGGAAAAGCAAACA
ATTA
>MOUSE
AATTGTTTCCATTATTAACTATGTCTAGTGAGTTCAGCCTTTAGTTGCTGTGTAAATCTATGGCTCCTAGTTGCATTGCTTTATT
TCAAACATCATTTCAAATTAGAGCTTAATGTGGTTTGAAGGTCTTGCCAGAATCCTCATCCTGCCTTTGTCTGAGGCTGGTTGAT
TACTTTTATCTTTTCCAGCTTATGAATGCCAATATGCTTAGAAGAAGGCAAACCCAGCCCCGAGACCATTTGTCATCACACATTG
ATTGT
>HUMAN
ATACTTGGTCTATTTAGAAAACAAACACATTTAATTGCTTCCATCTCTTAAATATGTCTAGTAAGTTCAGTTTTTAGTTGCTGTA
CAAATAAATTGGTCCTAGTTGCATTGCTTTCTTTCAAACATCATTTCAAATTAGGGCTTAATGTGGTTTGAACATTTTGCCAGAA
ATAGTCATCCTACTTTTGTCTGAGGGTGGTTGATTACATCTATCTTTTCCAGCTTACAAATGCCAATATATTTAGAGGAATGCAA
ACCCAACCCGGAGACCATTTGTCATCACACGTTGATTGTAACTCTATGTTTTATTATGGTGCGTAGGAGGAAAAGCAAACAAATA
```

```
           1                                                          60
  CHICKEN  ATACTGTGCCTGTTTAAATCACTAATATATTTTAATTGTTTCCATCTTTCAAAATATATC
    HUMAN  ATACTTGGTCTATTTAGAAAACAAACACATTT-AATTGCTTCCATCTCT-TAAATATGTC
    MOUSE                                  AATTGTTTCCATTATT--AACTATGTC
Consensus  atact..g.ct.ttta.a..ac.aa.a.attt.AATTGtTTCCATcttT..AAaTATgTC

           61                                                        120
  CHICKEN  TAACAAGTTCAATTTTTAGTTGTTGTTACACAAATAAACTGGTATTAGATGCATTGCTTT
    HUMAN  TAGTAAGTTCAGTTTTTAGTTGCTGT---ACAAATAAATTGGTCCTAGTTGCATTGCTTT
    MOUSE  TAGTGAGTTCAGCCTTTAGTTGCTGT---GTAAATCTATGGCTCCTAGTTGCATTGCTTT
Consensus  TAgtaAGTTCAgttTTTAGTTGcTGT...acAAATaaAttGgTccTAGtTGCATTGCTTT

           121                                                       180
  CHICKEN  CTTTCTTTCAAACATACTTTCAAATTACTGCTTAATGTGGTTTGAAGATTTTGCCAACAT
    HUMAN  CTTTC----AAACATCATTTCAAATTAGGGCTTAATGTGGTTTGAACATTTTGCCAGAAA
    MOUSE  ATTTC----AAACATCATTTCAAATTAGAGCTTAATGTGGTTTGAAGGTCTTGCCAGAA-
Consensus  cTTTC....AAACATcaTTTCAAATTAg.GCTTAATGTGGTTTGAAgaTtTTGCCAgaA.

           181                                                       240
  CHICKEN  AACCTTCCAGATCCTTTTCTTGTATGAGGGTGGTTGATTACATCTATCTTTT--GGTTTA
    HUMAN  TA--GTC---ATCCTACTTTTGTCTGAGGGTGGTTGATTACATCTATCTTTTCCAGCTTA
    MOUSE  -----TCCTCATCCTGCCTTTGTCTGAGGCTGGTTGATTACTTTTATCTTTTCCAGCTTA
Consensus  .a...TCc..ATCCT.cttTTGTcTGAGGgTGGTTGATTACaTcTATCTTTTccaGcTTA

           241                                                       300
  CHICKEN  CAAATGCCAATTTATTCCAAAGAATGCAAACCCT-----GGGACCATTTGTCATTACACT
    HUMAN  CAAATGCCAATATATTTAGAGGAATGCAAACCCAACCCGGAGACCATTTGTCATCACACG
    MOUSE  TGAATGCCAATATGCTTAGAAGAAGGCAAACCCAGCCCCGAGACCATTTGTCATCACACA
Consensus  caAATGCCAATaTatTtagAaGAAtGCAAACCCa.ccc.GaGACCATTTGTCATcACAC.

           301                                           354
  CHICKEN  TTGATTGTAGCTCACT---TCATTATTGTATGTAGGAGGAAAAGCAAACAATTA
    HUMAN  TTGATTGTAACTCTATGTTTTATTATGGTGCGTAGGAGGAAAAGCAAACAAATA
    MOUSE  TTGATTGT
Consensus  TTGATTGTa.ctc..t...t.attat.gt..gtaggaggaaaagcaaacaa.ta
```

Sup-Table 12 - SCL25A21. CIS of Chicken, Mouse and Human and an exonic segment of Gorilla; the corresponding alignments are shown. Consensus line (Chicken, Mouse and Human): capital red type = 3/3 identities; normal blue type = 2/3 identities; dot = less than two identities.

\>CHICKEN
TGCTTGGTCTCAGGTGATCCATAAAAAGGAATGAAGCCTTGGGAAGGTAAACCATCACCAGACTGGCTCGGTGTCTCAGCCTTCC
ATGACGGAAGCTCTTTGTTGAGAGTTGTTGGCAATTACAGCAGTTTCCAACTGAAGATAAAGAGCCAGAGTTCTCAGATAATGCC
CTCGATCACACACCTGATGACAGACTTCTGCATGCAAGTTTTTTCAGGTGCTATGTTTGCTCAGTAATCCTTCAGTGCCAATCTT
TTCTAAATGGTGAGCAAGTCCCGTCAGTAATGGACATCAGCCATTGAGCCTGTAATATTTGTCATATATCAGGTTACTGCTTTGT
TACTGCAGCCTGATGAATGGATGCTGTTATGCTTAGAGAAAATAAAGAAGTGCTATAAAT
\>MOUSE
CTCTTTGTTGAAAATTCTTGGCAGCGGCAGCAGTTTGCAGTTTCAGATAAGAGCTTCAGGTAGTGCTCTCCATCAGAGCCTTGAT
GACAGACTGTGGAGAGCGAGTTTTCTCAGGTGCTCTGCTTGCTCACTAATCCTTCAGTGCCGGCTCCTCCAGCAAGAGAGAGGGA
CCCATCAGTAATGGACATCAGCCATTAAGCTCCTAATATTTGTCATTTATCAGGTTACCTCTTTGTTACTCCCAAGTGATGAATG
GGTGCTGTGATGCCTTAAGAAAATAAAGCAATGCTATAAAT
\>HUMAN
TGCTTGGGCTCAGGTGATCCGTAAAGGGGGTGGGCTATGGAGAA**ATGGCGTACCCAGCCGCCTGCCTCGGTGGCTCAGCCTTTCA**
TGATCGCCACTCTTTGTTGAGAATTGTTGGCAGTGGCAGCGGTTTGCAGCTTCAGATAACAGCCGCCGTTCTCAGGTAATGCCTT
CGATCAGAGCCCTGATGACAGACTGTGGAGTGCAAGTTTTTTCAGGTGCTCTGTTTGCTCAGTAATCCTTCAGTGCCAATCTCCT
CCAAAGAGTGAGCAAGACCCATCCGTAATGGACATCAGCCATTAAGCTCATAGTATTTGTCGTCTATCAGGTTACTGCTTTGTTA
CTCTCCAGTGATGAATGGATGCTGTTATGGTTTAAGAAAATAAAGCAATGCTGTAAAT
\>GORILLA
ATGGCGTACCCAGCCGCCTGCCTCGGTGGCTCAGCCTTTCATGATCGCCACTCTTTGTTGAGAATTGTTGGCAGTGGCAGCGGTT
TGCAGCTTCAGATAACAGCCGCCGTTCTCAGGTTAT

```
           1                                                          60
  CHICKEN  TGCTTGGTCTCAGGTGATCCATAAAAAGGAATGAAGCCTTGGGAA-GGTAAACCATCACC
    MOUSE
    HUMAN  TGCTTGGGCTCAGGTGATCCGTAAAGGGGGTGGGCTATGGAGAAATGGCGTACC--CAGC
  GORILLA                                              ATGGCGTACC--CAGC
Consensus  ............................................a.ggcgtacc..cagc

           61                                                        120
  CHICKEN  AGACTGGCTCGGTGTCTCAGCCTTCCATGACGGAAGCTCTTTGTTGAGAGTTGTTGGCAA
    MOUSE                                      CTCTTTGTTGAAAATTCTTGGCAG
    HUMAN  CGCCTGCCTCGGTGGCTCAGCCTTTCATGATCGCCACTCTTTGTTGAGAATTGTTGGCAG
  GORILLA  CGCCTGCCTCGGTGGCTCAGCCTTTCATGATCGCCACTCTTTGTTGAGAATTGTTGGCAG
Consensus  cgcctgcctcggtggctcagcctttcatgatcgccaCTCTTTGTTGAgAaTTgTTGGCAg

           121                                                       180
  CHICKEN  TTACAGCAGTTTCCAACTGAAGATAAAGAGCCAGAGTTCTCAGATAATGCCCTCGATCAC
    MOUSE  CGGCAGCAGTTTGCAGTTTCAGATAA-GAGCT-------TCAGGTAGTGCTCTCCATCAG
    HUMAN  TGGCAGCGGTTTGCAGCTTCAGATAAC-AGCCGCCGTTCTCAGGTAATGCCTTCGATCAG
  GORILLA  TGGCAGCGGTTTGCAGCTTCAGATAAC-AGCCGCCGTTCTCAGGTTAT
Consensus  tggCAGCaGTTTgCAgcTtcAGATAA..AGCcgccgttcTCAGgTaaTGCccTCgATCAg

           181                                                       240
  CHICKEN  ACACCTGATGACAGACTTCTGCATGCAAGTTTTTTCAGGTGCTATGTTTGCTCAGTAATC
    MOUSE  AGCCTTGATGACAGACTGTGGAGAGCGAGTTTTCTCAGGTGCTCTGCTTGCTCACTAATC
    HUMAN  AGCCCTGATGACAGACTGTGGAGTGCAAGTTTTTTCAGGTGCTCTGTTTGCTCAGTAATC
  GORILLA
Consensus  AgcCcTGATGACAGACTgtgGagtGCaAGTTTTtTCAGGTGCTcTGtTTGCTCAgTAATC

           241                                                       300
  CHICKEN  CTTCAGTGCCAATCTTTTCTAAATGGTGAGCAAGTCCCGTCAGTAATGGACATCAGCCAT
    MOUSE  CTTCAGTGCCGG-CTCCTCCAGCAAGAGAGAGGGACCCATCAGTAATGGACATCAGCCAT
    HUMAN  CTTCAGTGCCAATCTCCTCCAAAGAGTGAGCAAGACCCATCCGTAATGGACATCAGCCAT
  GORILLA
```

```
Consensus  CTTCAGTGCCaatCTccTCcAaa.aGtGAGcaaGaCCCaTCaGTAATGGACATCAGCCAT

           301                                                       360
  CHICKEN  TGAGCCTGTAATATTTGTCATATATCAGGTTACTGCTTTGTTACTGCAGCCTGATGAATG
    MOUSE  TAAGCTCCTAATATTTGTCATTTATCAGGTTACCTCTTTGTTACTCCCAAGTGATGAATG
    HUMAN  TAAGCTCATAGTATTTGTCGTCTATCAGGTTACTGCTTTGTTACTCTCCAGTGATGAATG
  GORILLA
Consensus  TaAGCtc.TAaTATTTGTCaT.TATCAGGTTACtgCTTTGTTACTccc.agTGATGAATG

           361                                     401
  CHICKEN  GATGCTGTTATGCTTAGAGAAAATAAAGAAGTGCTATAAAT
    MOUSE  GGTGCTGTGATGCCTTAAGAAAATAAAGCAATGCTATAAAT
    HUMAN  GATGCTGTTATGGTTTAAGAAAATAAAGCAATGCTGTAAAT
  GORILLA
Consensus  GaTGCTGTtATGctTtaAGAAAATAAAGcAaTGCTaTAAAT
```

Sup-Table 13 - SCL25A21. CIS of Chicken, Mouse and Human and exonic segments of Anas and Zonotrichia; the corresponding alignments are shown. Consensus line: capital red type = 5/5 identities; normal blue type = 4/5 or 3/5 identities.

```
>CHICKEN
TGCCAATAAAATGGTTAACAGATGAACTTTGGGGGCGATTTATATTGTTCATGCATTCCCAGACATCTTGTTTCAAGCATATATT
TTTACCTTAGTGTTATTGAGACATCTG
>MOUSE
TGCCAGAACAATGGTTAACAGATGAACTGCTGGGCAACTGGGTCTGGTGGATGTGTCCCAGACATCTTGTAGCAAGTAGATTTTA
CCTTAAAATTATTGAGACATATG
>HUMAN
TGCCAGAACAATGGTTAACAGATGAACTTTTGGGCAACTGAATATGGTGTATGTGTCCCAGACATCTTGTTTCAAGTAGATTTTA
CCTTAAAATTATTGAGACATATG
>ANAS
TGCCAATACAATGGTTAACAGATGAACTTTTGGGGCAAATTTATATTGTTCGTGCATTCCCAGACATCTTGTTTCAAGCATATAT
TTTTACCTTAGAGTTATTGAGACATCTG
>ZONOTRICHIA
TGCCAATACAATGGTTAACAGATGAACTTTTGGGGCAAATTTATATTGCTCATGTGTTCCCAGACATCTTGTTTCAAGCATATAT
TTTTATCTTAGCATTATTGAGACATCTG

              1                                                          60
      CHICKEN TGCCAATAAAATGGTTAACAGATGAACTTTGGGG-GCGATTTATATTGTTCATGCATTCC
        MOUSE TGCCAGAACAATGGTTAACAGATGAACTGCTGGG-CAACTGGGTCTGGTGGATGTGT-CC
        HUMAN TGCCAGAACAATGGTTAACAGATGAACTTTTGGG-CAACTGAATATGGTGTATGTGT-CC
         ANAS TGCCAATACAATGGTTAACAGATGAACTTTTGGGGCAAATTTATATTGTTCGTGCATTCC
  ZONOTRICHIA TGCCAATACAATGGTTAACAGATGAACTTTTGGGGCAAATTTATATTGCTCATGTGTTCC
    Consensus TGCCAatAcAATGGTTAACAGATGAACTtttGGGgcaaaTttaTaTtGttcaTGtgTtCC

              61                                                  113
      CHICKEN CAGACATCTTGTTTCAAGCATATATTTTTACCTTAGTGTTATTGAGACATCTG
        MOUSE CAGACATCTTGTAGCAAGTAGAT---TTTACCTTAAAATTATTGAGACATATG
        HUMAN CAGACATCTTGTTTCAAGTAGAT---TTTACCTTAAAATTATTGAGACATATG
         ANAS CAGACATCTTGTTTCAAGCATATATTTTTACCTTAGAGTTATTGAGACATCTG
  ZONOTRICHIA CAGACATCTTGTTTCAAGCATATATTTTTATCTTAGCATTATTGAGACATCTG
    Consensus CAGACATCTTGTttCAAGcAtATattTTTAcCTTAgaaTTATTGAGACATcTG
```

Sup-Table 14 - SCL25A21. CIS of Chicken, Mouse and Human and an exonic segments of Physeter; the corresponding alignments are shown. Consensus line: capital red type = 4/4 identities; normal blue type = 3/4 identities; dot = ≤ 2/4 identities. Chicken/Mouse consensus at the beginning and Chicken/Human consensus at the end of the alignments are indicated in normal black type.

>CHICKEN
AGTAGGGTTCAGAAGCAATCTGTTGTGAATTCTTCAGCCAATTAGCTGAGAGAGTTACCACTGCTTATAATGCACAGAATGTGGA
GGCTCTCTTGCCGTTGCACTGAGCTGGATTGTTAATAGCAGGAAGCTAGCACTTCTCAGTACAGTGGATCAGCTACATGTGAAAA
TAAATGTAAAAAACCTGGAGGGGAATGGCAGCAGACTGAGATTAGCCTTTATTTAATTTAGCATTTGGGGGTCCAACACACAAAT
GAGCATTTTAGTAGGCTTCATGGTGGAAGTTGCTGTCATTTGTAAAATATTCTGGCCAATTAGCTAATAGTGTGCTTGGATTTCT
CCTGTTTTGATACAGTAATACTAAGTACATTGTGAAGCCCAATTATACAAATCATCCCCATATGCCATACCAGTCTTTCTTTATG
AACTGTGTTTATAAATCCTGTGTGAGGAAATGTGTACTTCAGCAATTTAGACC**ATGTGTAAAAACATAGCCAATAGGATTGTCAA**
GAAAACAAGTTCCAGCATAGGAAATCCTAATGTGTTCCGCTAAAATAACATGAGTGTGTTTTGTTGTGTGAGTTTTCTGATTAAA
TGCATATTACTTTATAGTGCTTAACCTGCATAGGTCCTTCCGTTTCCCTT
>MOUSE
AGTAAGATTCAGGAACAATGGGTTGTAAATTCTTGAGCCAATTAGCCCAGTGACTTACTACTGTTTCTAATGCACAGAATGTCAC
AGGCTGTTTTGCAGCCGCCCCAAGTCGGTTTGTTAATAACAGGAAGCTAGCACTTCTCAGTACAGTGGATCAAGCTACACGTGAA
AATAAATGCAAAAAATCTGGAGGGGAATAGCAGCAGACCAGGAGTAGCCTTGATTTAATTTAACATTCGGGGTCCGCACACATGA
GCGTTTTAGTAGGCTTCATGGTGGAAGTTGCTGTCATTTGTAAAATATTCTGGCCAATTAGCTAATAGTGTGCTTGGATTTCTCC
TGTTTTGATACAGTAATACTAAGTACATTGTGAAGCCCAATTATACAAATCATCCCCATATGCCGTACCAGTCTTTCTTTATGAA
CTGTGTTTATAAATCCTGTGTGAGGAAATGTGTACTTCAGCAATTTAGACC**ATGTGTAAAAACATAGCCAATGGGATTGTCAAGA**
AAACAAGTTCCAGCCTAGGAAATCCTAATGTGCTCGGCTGGAATAACAGGAGGGTG
>HUMAN
TTGTAAATTCTTCAGCCAATTACTCCAGTGACTTACTACTGTTTATAATGCACAGAATGTCACAGGCTGTTTTGCAGCCGCCCCA
AGTTGGTTTGTTAATAACAGGAAGCTAGCACTTCTCAGTACAGTGGATCAGCTACACGTGAAAATAAATGCAAAAACCTGGCGG
GCAATAGCAGCAGACCGCGAGTAGCCTTGATTTAATTTAACATTTGGGGGTCCACACACAAATGAGCATTTTAGTAGGCTTCATG
GTGGAAGTTGCTGTCATTTGTAAAATATTCTGGCCAATTAGCTAATAGTGTGCTTGGATTTCTCCTGTTTTGATACAGTAATACT
AAGTACATTGTGAAGCCCAATTATACAAATCATCCCCATATGCCATACCAGTCTTTCTTTATGAACTGTGTTTATAAATCCTGTG
TGAGGAAATGTGTACTTCAGCAATTTAGACC**ATGTGTAAAAACATAGCCAATGGGATTGTCAAGAAAACAAGTTCCAGCCTAGGA**
AATCCTAATGTGTTCCGCTGGAATAACAGGAGGGTGCCGGGAGTGCAATGCTCTGCTTAAATACATATTGCTTTATAGTGTTTAA
CTGATTTAGGGGCTTCCGCTCCCCTT
>PHYSETER
ATGTGTAAAAACATAGCCAATGGGATTGTCAAGAAAACAAGTTCCAGCCTAGGAAATCCTAATGTGTTCCGCTGGAATAACAGGA
G

```
             1                                                          60
   CHICKEN   AGTAGGGTTCAGAAGCAATCTGTTGTGAATTCTTCAGCCAATTAGCTGAGAGAGTTACCA
     HUMAN                         TTGTAAATTCTTCAGCCAATTACTCCAGTGACTTACTA
     MOUSE   AGTAAGATTCAGGAACAATGGGTTGTAAATTCTTGAGCCAATTAGCCCAGTGACTTACTA
  PHYSETER
 Consensus   agta.g.ttcag.a.caat..gttgt.aattctt.agccaatta....ag.ga.ttac.a

             61                                                        120
   CHICKEN   CTGCTTATAATGCACAGAATGTGG-AGGCTCTCTTGCCGTTGCACTGAGCTGGATTGTTA
     HUMAN   CTGTTTATAATGCACAGAATGTCACAGGCTGTTTTGCAGCCGCCCCAAGTTGGTTTGTTA
     MOUSE   CTGTTTCTAATGCACAGAATGTCACAGGCTGTTTTGCAGCCGCCCCAAGTCGGTTTGTTA
  PHYSETER
 Consensus   ctg.tt.taatgcacagaatgt...aggct.t.ttgc.g..gc.c..ag..gg.ttgtta

             121                                                       180
   CHICKEN   ATAGCAGGAAGCTAGCACTTCTCAGTACAGTGGATCA-GCTACATGTGAAAATAAATGTA
     HUMAN   ATAACAGGAAGCTAGCACTTCTCAGTACAGTGGATCA-GCTACACGTGAAAATAAATGCA
     MOUSE   ATAACAGGAAGCTAGCACTTCTCAGTACAGTGGATCAAGCTACACGTGAAAATAAATGCA
  PHYSETER
 Consensus   ata.caggaagctagcacttctcagtacagtggatca.gctaca.gtgaaaataaatg.a

             181                                                       240
```

```
  CHICKEN  AAAAACCTGGAGGGGAATGGCAGCAGACTGAGATTAGCCTTTATTTAATTTAGCATTTGG
    HUMAN  AAAAACCTGGCGGGCAATAGCAGCAGACCGCGAGTAGCCTTGATTTAATTTAACATTTGG
    MOUSE  AAAAATCTGGAGGGGAATAGCAGCAGACCAGGAGTAGCCTTGATTTAATTTAACATTCGG
 PHYSETER
Consensus  aaaaa.ctgg.ggg.aat.gcagcagac...ga.tagcctt.atttaattta.catt.gg

           241                                                      300
  CHICKEN  GGGTCCAACACACAAATGAGCATTTTAGTAGGCTTCATGGTGGAAGTTGCTGTCATTTGT
    HUMAN  GGGTCCA-CACACAAATGAGCATTTTAGTAGGCTTCATGGTGGAAGTTGCTGTCATTTGT
    MOUSE  GG-TCCG---CACACATGAGCGTTTTAGTAGGCTTCATGGTGGAAGTTGCTGTCATTTGT
 PHYSETER
Consensus  gg.tcc....caca.atgagc.ttttagtaggcttcatggtggaagttgctgtcatttgt

           301                                                      360
  CHICKEN  AAAATATTCTGGCCAATTAGCTAATAGTGTGCTTGGATTTCTCCTGTTTTGATACAGTAA
    HUMAN  AAAATATTCTGGCCAATTAGCTAATAGTGTGCTTGGATTTCTCCTGTTTTGATACAGTAA
    MOUSE  AAAATATTCTGGCCAATTAGCTAATAGTGTGCTTGGATTTCTCCTGTTTTGATACAGTAA
 PHYSETER
Consensus  aaaatattctggccaattagctaatagtgtgcttggatttctcctgttttgatacagtaa

           361                                                      420
  CHICKEN  TACTAAGTACATTGTGAAGCCCAATTATACAAATCATCCCCATATGCCATACCAGTCTTT
    HUMAN  TACTAAGTACATTGTGAAGCCCAATTATACAAATCATCCCCATATGCCATACCAGTCTTT
    MOUSE  TACTAAGTACATTGTGAAGCCCAATTATACAAATCATCCCCATATGCCGTACCAGTCTTT
 PHYSETER
Consensus  tactaagtacattgtgaagcccaattatacaaatcatccccatatgcc.taccagtcttt

           421                                                      480
  CHICKEN  CTTTATGAACTGTGTTTATAAATCCTGTGTGAGGAAATGTGTACTTCAGCAATTTAGACC
    HUMAN  CTTTATGAACTGTGTTTATAAATCCTGTGTGAGGAAATGTGTACTTCAGCAATTTAGACC
    MOUSE  CTTTATGAACTGTGTTTATAAATCCTGTGTGAGGAAATGTGTACTTCAGCAATTTAGACC
 PHYSETER
Consensus  ctttatgaactgtgtttataaatcctgtgtgaggaaatgtgtacttcagcaatttagacc

           481                                                      540
  CHICKEN  ATGTGTAAAAACATAGCCAATAGGATTGTCAAGAAAACAAGTTCCAGCATAGGAAATCCT
    HUMAN  ATGTGTAAAAACATAGCCAATGGGATTGTCAAGAAAACAAGTTCCAGCCTAGGAAATCCT
    MOUSE  ATGTGTAAAAACATAGCCAATGGGATTGTCAAGAAAACAAGTTCCAGCCTAGGAAATCCT
 PHYSETER  ATGTGTAAAAACATAGCCAATGGGATTGTCAAGAAAACAAGTTCCAGCCTAGGAAATCCT
Consensus  ATGTGTAAAAACATAGCCAATgGGATTGTCAAGAAAACAAGTTCCAGCcTAGGAAATCCT

           541                                                      600
  CHICKEN  AATGTGTTCCGCTAAAATAACATGAGTGTGTTTTGTTGTGTGAGTTTTCTGATTAAATGC
    HUMAN  AATGTGTTCCGCTGGAATAACAGGAGGGTGCCGGGA-GTGCAA-TGCTCTGCTTAAATAC
    MOUSE  AATGTGCTCGGCTGGAATAACAGGAGGGTG------------------------------
 PHYSETER  AATGTGTTCCGCTGGAATAACAGGAG----------------------------------
Consensus  AATGTGtTCcGCTggAATAACAgGAG.gtg....g..gtg..a.t..tctg.ttaaat.c

           601                                           647
  CHICKEN  ATATTACTTTATAGTGCTTAACCTGCATAGGTCCTTCCGTTTCCCTT
    HUMAN  ATATTGCTTTATAGTGTTTAACTGATTTAGGGGCTTCCGCTCCCCTT
    MOUSE  -----------------------------------------------
 PHYSETER  -----------------------------------------------
Consensus  atatt.ctttatagtg.ttaac.....tagg..cttccg.t.ccctt
```

<u>Sup-Table 15</u> - SCL25A25. Conserved genomic sequences of Zebrafish (slc25a25-A), Chicken, Mouse and Human and the corresponding alignments. Consensus line: capital red type = 4/4 identities; normal blue type = 3/4 identities; dot = ≤ 2/4 identities.

```
>ZEBRAFISH (a25-A)
ATGTTGTGCCTGTGCCTTTACGTGCCTGTTCATAATTCTGACCAGATTGAAGTGGAGTATTTTGAGTCGAATGGATTACCGTCCG
AGCTGAAGTCTCTCAAGTCTCTGAGTGTCCTTCTGCCGTCACAAGAGTTCTCCACATACCGAAGATGGAGGAAG
>CHICKEN
ATGCTCTGCCTCTGTCTCTATGTGCCGGTGCTGGGGCAGTCGCAGGCAGAGTTTGAGTACTTCGAGTCGAAGGGGCTGCCGGCCG
AGCTCAAGTCTATCTTCCGCCTCAGCCTCTTCATCCCCTCCCAGGAGTTCTCCACCTACCGCCAGTGGAAGCAG
>MOUSE
ATGCTCTGCCTGTGCCTGTATGTGCCCATCGCCGGGGCGGCTCAGACTGAGTTCCAGTACTTTGAGTCCAAGGGGCTTCCTGCCG
AGCTGAAATCCATCTTCAAACTCAGTGTCTTTATCCCCTCTCAAGAGTTCTCCACATACCGCCAATGGAAGCAG
>HUMAN
ATGCTCTGTCTGTGCCTGTATGTGCCGGTCATCGGGGAAGCCCAGACCGAGTTCCAGTACTTTGAGTCGAAGGGGCTCCCTGCCG
AGCTGAAGTCCATTTTCAAGCTCAGTGTCTTCATCCCCTCCCAGGAATTCTCCACCTACCGCCAGTGGAAGCAG
```

```
            1                                                          60
 ZEBRAFISH  ATGTTGTGCCTGTGCCTTTACGTGCCTGTTCATAATTCTGACCAGATTGAAGTGGAGTAT
   CHICKEN  ATGCTCTGCCTCTGTCTCTATGTGCCGGTGCTGGGGCAGTCGCAGGCAGAGTTTGAGTAC
     MOUSE  ATGCTCTGCCTGTGCCTGTATGTGCCCATCGCCGGGGCGGCTCAGACTGAGTTCCAGTAC
     HUMAN  ATGCTCTGTCTGTGCCTGTATGTGCCGGTCATCGGGGAAGCCCAGACCGAGTTCCAGTAC
 Consensus  ATGcTcTGcCTgTGcCT.TAtGTGCC.gT.c..ggg...gccCAGac.GAgtT..AGTAc

            61                                                        120
 ZEBRAFISH  TTTGAGTCGAATGGATTACCGTCCGAGCTGAAGTCTCTCAAGTCTCTGAGTGTCCTTCTG
   CHICKEN  TTCGAGTCGAAGGGGCTGCCGGCCGAGCTCAAGTCTATCTTCCGCCTCAGCCTCTTCATC
     MOUSE  TTTGAGTCCAAGGGGCTTCCTGCCGAGCTGAAATCCATCTTCAAACTCAGTGTCTTTATC
     HUMAN  TTTGAGTCGAAGGGGCTCCCTGCCGAGCTGAAGTCCATTTTCAAGCTCAGTGTCTTCATC
 Consensus  TTtGAGTCgAAgGGgcT.CC.gCCGAGCTgAAgTC.aTcttc...CTcAGtgTCtT.aTc

            121                                    159
 ZEBRAFISH  CCGTCACAAGAGTTCTCCACATACCGAAGATGGAGGAAG
   CHICKEN  CCCTCCCAGGAGTTCTCCACCTACCGCCAGTGGAAGCAG
     MOUSE  CCCTCTCAAGAGTTCTCCACATACCGCCAATGGAAGCAG
     HUMAN  CCCTCCCAGGAATTCTCCACCTACCGCCAGTGGAAGCAG
 Consensus  CCcTC.CA.GAgTTCTCCAC.TACCGcca.TGGAaGcAG
```

Percent Identity Matrix

	ZEBRAFISH	CHICKEN	MOUSE	HUMAN
ZEBRAFISH	-----	64.15	68.55	65.41
CHICKEN	64.15	-----	78.62	83.02
MOUSE	68.55	78.62	-----	87.42
HUMAN	65.41	83.02	87.42	-----

<u>Sup-Table 16</u> - SCL25A25. Conserved genomic sequences of Chicken, Mouse and Human and the corresponding alignments. Consensus line: capital red type = 3/3 identities; normal blue type = 2/3 identities.

>CHICKEN
TTTGCCTGTGTGTCAGAATAAGGACGGGACACTTCTGGGGTCCTGTCACCTAGTAAGTAT
>MOUSE
TTTGTCTGTCTGTCAGAATACGGACGGGCCACTTCTGGGGCCCTGTCACCTAGTAAGTAT
>HUMAN
TTTGTCTGTCTGTCAGAATACGAACGGGCCATTTCTGGGGCCCTGTCACCTAGTAAGTAT

	1 … 60
CHICKEN	TTTGCCTGTGTGTCAGAATAAGGACGGGACACTTCTGGGGTCCTGTCACCTAGTAAGTAT
MOUSE	TTTGTCTGTCTGTCAGAATACGGACGGGCCACTTCTGGGGCCCTGTCACCTAGTAAGTAT
HUMAN	TTTGTCTGTCTGTCAGAATACGAACGGGCCATTTCTGGGGCCCTGTCACCTAGTAAGTAT
Consensus	TTTGtCTGTcTGTCAGAATAcGgACGGGcCAcTTCTGGGGcCCTGTCACCTAGTAAGTAT

Sup-Table 17 - SCL25A25. Blue boxes: transcript components which are actually expressed in the different variants. See Text for details.

HUMAN													
Variant	2	X1		3	5				1	X2		X3	X4
Exon 1 optional													
Exon 2 optional													
First conserved													
Exon 3 constant													
Exon 4 constant													
Second conserved													
7 Exons constant													
MOUSE													
Variant	2	1		3	X3	X1	X4		4	X2			
Exon 1 optional													
Exon 2 optional													
First conserved													
Exon 3 constant													
Exon 4 constant													
Second conserved													
7 Exons constant													
CHICKEN													
Variant	X4	X5		X3	X1	X7			X2	X6			
Exon 1 optional													
Exon 2 optional													
First conserved													
Exon 3 constant													
Exon 4 constant													
Second conserved													
6/7 Exons constant	6 ex	7 ex		7 ex	7 ex	6 ex			7 ex	6 ex			
ZEBRAFISH													
Variant									a				
Exon 1 optional													
Exon 2 optional													
First conserved													
Exon 3 constant													
Exon 4 constant													
Second conserved													
7 Exons constant													

Sup-Table 18 – SLC25A29. A conserved DNA sequence of Chicken and Human and the corresponding alignment. Consensus line: capital red type = nucleotide identity in the two sequences; dot = different nucleotides in the two sequences.

>CHICKEN
GATGTAATGTGTAAAATTGCTTTTTTGCTTTTAACTGCCTTTACTTTCTGAGCTAGGGGCTGTCTTCATTAACAGCAAAAGTGTT
ACTTTGTTGTAGATGAGAGATAAGCCTCATCACGGGAGAGTTACCTAGTAAGTATAGCCCAGGTGAAC
>HUMAN
GATGTGATGTTGAAATTTTGTGTTGCTTTAACCGCCTTTACTTTCTGAGCTAGGGGCTGTCTCCATCCATAGCAAAAGTGTAACT
TTGTTGTAGATGGGCGTAAGCCTCACCCTGGAGAGCGCTAGTAAGTACAGACCAGGTGAAC

```
          1                                                          60
  CHICKEN   GATGTAATGTGTAAAATTGCTTTTTTGCTTTTAACTGCCTTTACTTTCTGAGCTAGGGGC
    HUMAN   GATGTGATGTTGAAATTT--TGTGTTGCTTT-AACCGCCTTTACTTTCTGAGCTAGGGGC
Consensus   GATGT.ATGT..AAA.TT..T.T.TTGCTTT.AAC.GCCTTTACTTTCTGAGCTAGGGGC

            61                                                        120
  CHICKEN   TGTCTTCATTAACAGCAAAAGTGTTACTTTGTTGTAGATGAGAGATAAGCCTCATCACGG
    HUMAN   TGTCTCCATCCATAGCAAAAGTGTAACTTTGTTGTAGATGGGCG-TAAGCCTCA-CCCTG
Consensus   TGTCT.CAT..A.AGCAAAAGTGT.ACTTTGTTGTAGATG.G.G.TAAGCCTCA.C.C.G

            121                             153
  CHICKEN   GAGAGTTACCTAGTAAGTATAGCCCAGGTGAAC
    HUMAN   GAGAGCG--CTAGTAAGTACAGACCAGGTGAAC
Consensus   GAGAG....CTAGTAAGTA.AG.CCAGGTGAAC
```

<u>Sup-Table 19</u> – SLC25A36. CIS of Zebrafish (slc25a36-A), Chicken, Mouse and Human and the corresponding alignments. Consensus line: capital red type = 4/4 identities; normal blue type = 3/4 identities; dot = ≤ 2/4 identities.

```
>ZEBRAFISH (slc25a3-A)
CTGCTTGTGATTTTGGTACAGAAAAGCCTGTGTCTATTTTAGGCCTTTACTGTAGCTTTCACCCTGCGTGAGGGTCTGTGAGCCA
GCCTGCTCTGCCCCCCATATCTGCAGGCTTCGTCCTGCAGCTGTGAGGA
>CHICKEN
CTGCTTGTTGTTATGATACAGAAAAGCCTGTGTCTATTTTAGGCATTTACTGTACATTTCTCCCGTGAAAAGAGTGAGATCGTGT
CATCTCATGCTCCCCATCCGCAGGTCACTTCCTGCAGAAATATGGA
>MOUSE
CTGCTTGTTGTTATGATACAGAAAGCCTGTGTCTATTTTAGGCATTTACTGTACATTTCTCCCGAGAAAAGAGTGAGATCGTGTC
ATCTCATGCTCCCCATCCGCAGGTCACTTCCTGTAGAAATATGGA
>HUMAN
CTGCTTGTTGTTATGATACAGAAAAGCCTGTGTCTATTTTAGGCATTTACTGTACATTTCTCCCGAGAAAAGAGTGAGATCGTGT
CATCTCATGCTCCCCATCCGCAGGTCACTTCCTGTAGAAATATGGA
```

```
            1                                                          60
ZEBRAFISH   CTGCTTGTGATTTTGGTACAGAAAAGCCTGTGTCTATTTTAGGCCTTTACTGTAGCTTTC
  CHICKEN   CTGCTTGTTGTTATGATACAGAAAAGCCTGTGTCTATTTTAGGCATTTACTGTACATTTC
    MOUSE   CTGCTTGTTGTTATGATACAGAAA-GCCTGTGTCTATTTTAGGCATTTACTGTACATTTC
    HUMAN   CTGCTTGTTGTTATGATACAGAAAAGCCTGTGTCTATTTTAGGCATTTACTGTACATTTC
Consensus   CTGCTTGTtgTTaTGaTACAGAAAaGCCTGTGTCTATTTTAGGCaTTTACTGTAcaTTTC

            61                                                        120
ZEBRAFISH   ACCCTGCGTGAGGGTCTGTGAGCCAGCCTGCTC-TGCCCCCCATATCTGCAGGCTTCGTC
  CHICKEN   TCCCGTGAAAAGAGT--GAGATCGTGTCATCTCATGCTCCCCAT--CCGCAGGTCACTTC
    MOUSE   TCCCGAGAAAAGAGT--GAGATCGTGTCATCTCATGCTCCCCAT--CCGCAGGTCACTTC
    HUMAN   TCCCGAGAAAAGAGT--GAGATCGTGTCATCTCATGCTCCCCAT--CCGCAGGTCACTTC
Consensus   tCCCg.gaaaAGaGT..GaGAtCgtGtCatCTCaTGCtCCCCAT..CcGCAGGtcaCtTC

            121           135
ZEBRAFISH   CTGCAGCTGTGAGGA
  CHICKEN   CTGCAGAAATATGGA
    MOUSE   CTGTAGAAATATGGA
    HUMAN   CTGTAGAAATATGGA
Consensus   CTG.AGaaaTatGGA
```

Percent Identity Matrix

	ZEBRAFISH	CHICKEN	MOUSE	HUMAN
ZEBRAFISH	-----	64.89	63.85	64.12
CHICKEN	64.89	-----	98.46	98.47
MOUSE	63.85	98.46	-----	100.00
HUMAN	64.12	98.47	100.00	-----
